# Supplementary material for: New Benzothiazole–Monoterpenoid Hybrids as Multifunctional Molecules with Potential Applications in Cosmetics
Source: Molecules. 2025 Jan 31;30(3):636. doi: 10.3390/molecules30030636 (PMC11820011; doi:10.3390/molecules30030636)
Supplement: Supplementary file 1 [file molecules-30-00636-s001.zip › molecules-3365832-supplementary.pdf]

Supplementary Materials

New benzothiazole-monoterpenoid hybrids as multifunctional molecules with potential applications in cosmetics

Desislava Kirkova [1], Yordan Stremski [2,*], Maria Bachvarova [2], Mina Todorova [2], Bogdan Goranov [3], Stela Statkova-Abeghe [2] and Margarita Docheva [1]

[1] Agricultural academy, Tobacco and Tobacco Products Institute, 4108 Markovo, Bulgaria; desislavaa894@gmail.com (D.K.), margarita_1980@abv.bg (M.D.);
[2] Department of Organic Chemistry, University of Plovdiv "Paisii Hilendarski", 24 Tsar Asen Str., 4000 Plovdiv, Bulgaria; stab@uni-plovdiv.bg (S.S.-A.), bychvarova@uni-plovdiv.bg (M.B.), minatodorova@uni-plovdiv.bg (M.T.);
[3] University of Food Technologies, 26 Maritza Boulevard, Department of Microbiology and Biotechnology, 4002 Plovdiv, Bulgaria; b_goranov@uft-plovdiv.bg (B.G.);
***Correspondence:** stremski@uni-plovdiv.net (Y.S.); Tel.: +359-32-261-346

Table of Contents:

Figure S1 ^{1}H-NMR spectrum of compound **4a**, **page 2**
Figure S2 ^{13}C-NMR spectrum of compound **4a**, **page 3**
Figure S3 IR spectrum of compound **4a**, **page 3**
Figure S4 MS spectrum of compound **4a**, **page 4**
Figure S5 ^{1}H-NMR spectrum of compound **4b**, **page 4-5**
Figure S6 ^{13}C-NMR spectrum of compound **4b**, **page 5**
Figure S7 IR spectrum of compound **4b**, **page 6**
Figure S8 MS spectrum of compound **4b**, **page 6**
Figure S9 ^{1}H-NMR spectrum of compound **4c**, **page 7**
Figure S10 ^{13}C-NMR spectrum of compound **4c**, **page 8**
Figure S11 IR spectrum of compound **4c**, **page 8**
Figure S12 MS spectrum of compound **4c**, **page 9**
Figure S13 ^{1}H-NMR spectrum of compound **5a**, **page 9-10**
Figure S14 ^{13}C-NMR spectrum of compound **5a**, **page 10**
Figure S15 IR spectrum of compound **5a**, **page 11**
Figure S16 MS spectrum of compound **5a**, **page 11**
Figure S17 ^{1}H-NMR spectrum of compound **5b**, **page 12**
Figure S18 ^{13}C-NMR spectrum of compound **5b**, **page 13**
Figure S19 IR spectrum of compound **5b**, **page 13**
Figure S20 MS spectrum of compound **5b**, **page 14**
Figure S21 ^{1}H-NMR spectrum of compound **5c**, **page 14-15**
Figure S22 ^{13}C-NMR spectrum of compound **5c**, **page 15**
Figure S23 IR spectrum of compound **5c**, **page 16**
Figure S24 MS spectrum of compound **5c**, **page 16**
Figure S25 ^{1}H-NMR spectrum of compound **6**, **page 17**
Figure S26 ^{13}C-NMR spectrum of compound **6**, **page 18**
Figure S27 IR spectrum of compound **6**, **page 18**
Figure S28 MS spectrum of compound **6**, **page 19**
Figure S29 ^{1}H-NMR spectrum of compound **7**, **page 19-20**
Figure S30 ^{13}C-NMR spectrum of compound **7**, **page 20**
Figure S31 IR spectrum of compound **7**, **page 21**
Figure S32 MS spectrum of compound **7**, **page 21**
Figure S33 - Table S1 GC-MS/MS analysis of thyme oil, **page 22-23**
Figure S34 - Table S2 GC-MS/MS analysis of oregano oil, **page 23-24**

Figure S1 ^{1}H-NMR spectrum of **4a** - DMSO-d6, 80 °C, 600 MHz

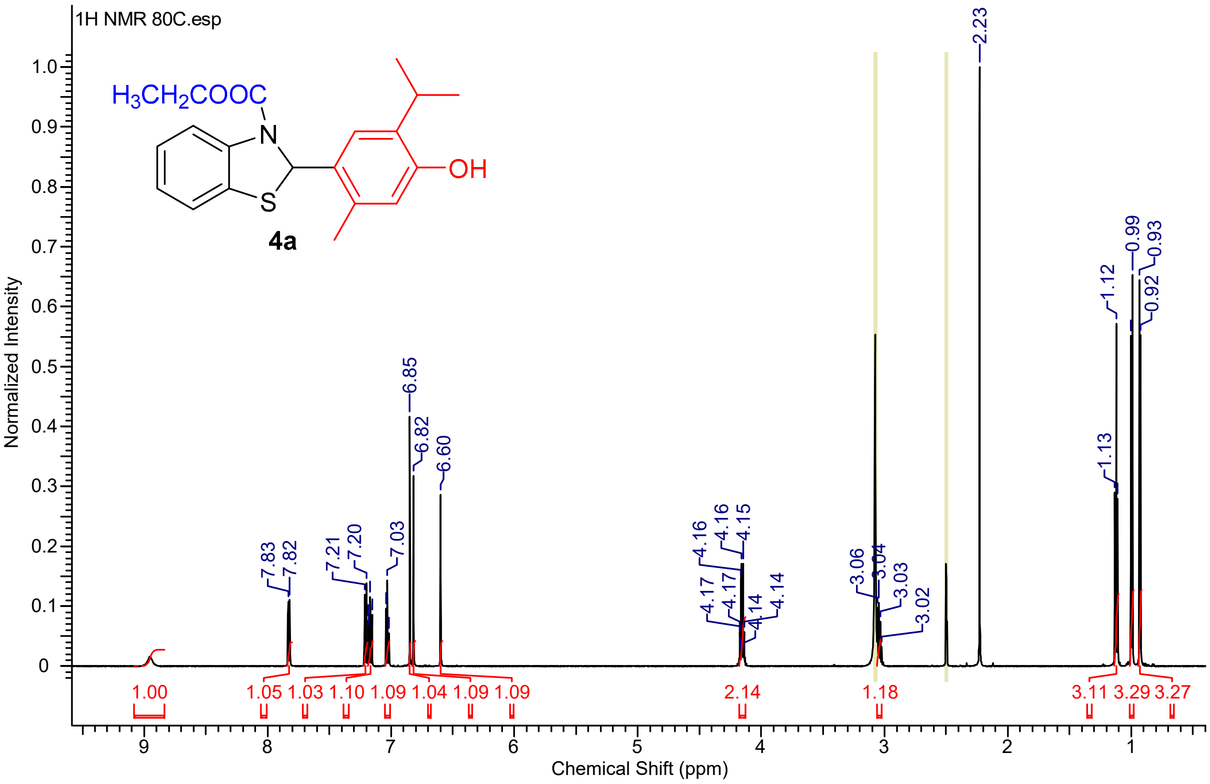

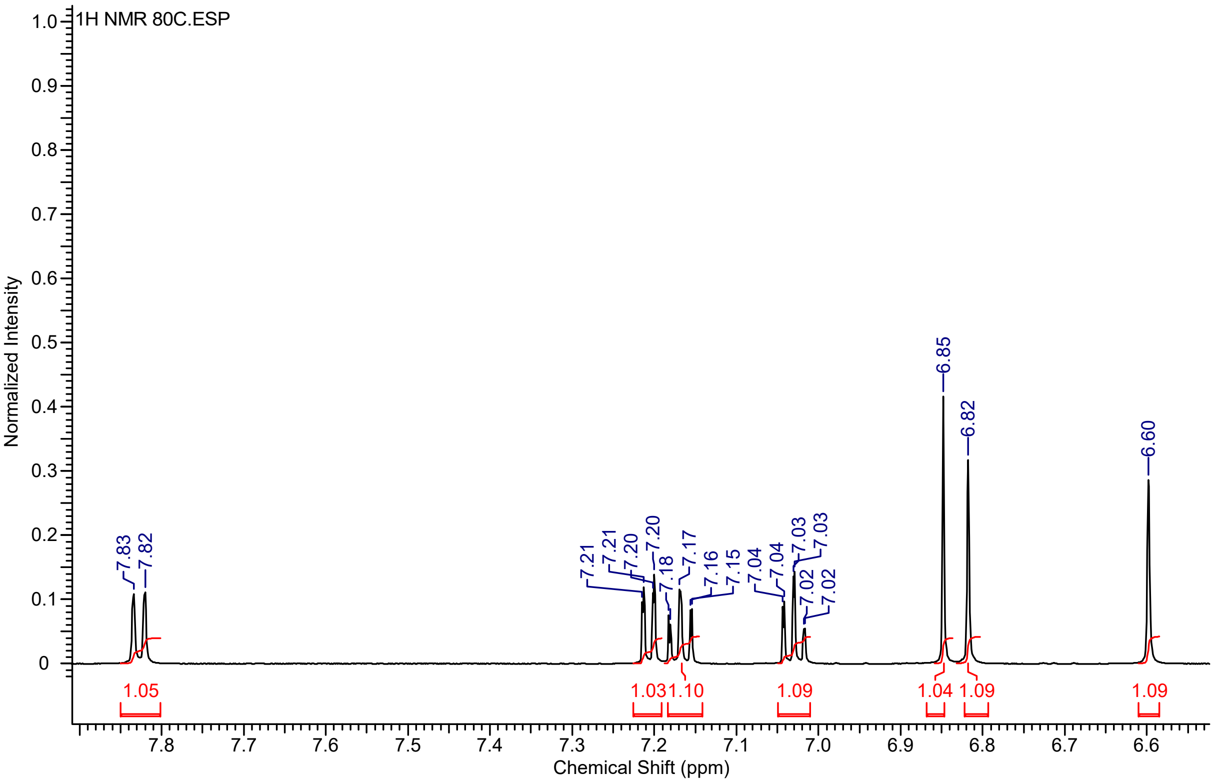

Figure S2 ^{13}C-NMR spectrum of **4a** - DMSO-d$_6$, 80 ºC, 150 MHz

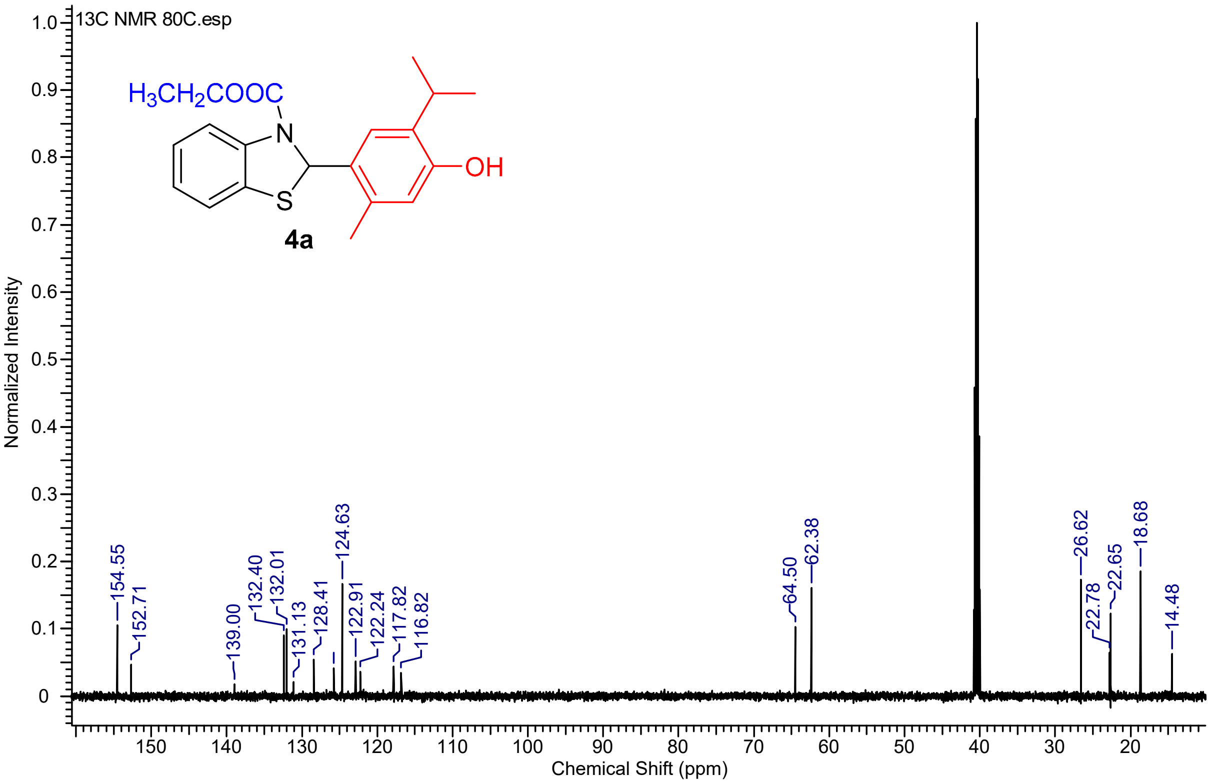

Figure S3 FT-IR spectrum of compound **4a**, KBr tablet, cm^{-1}

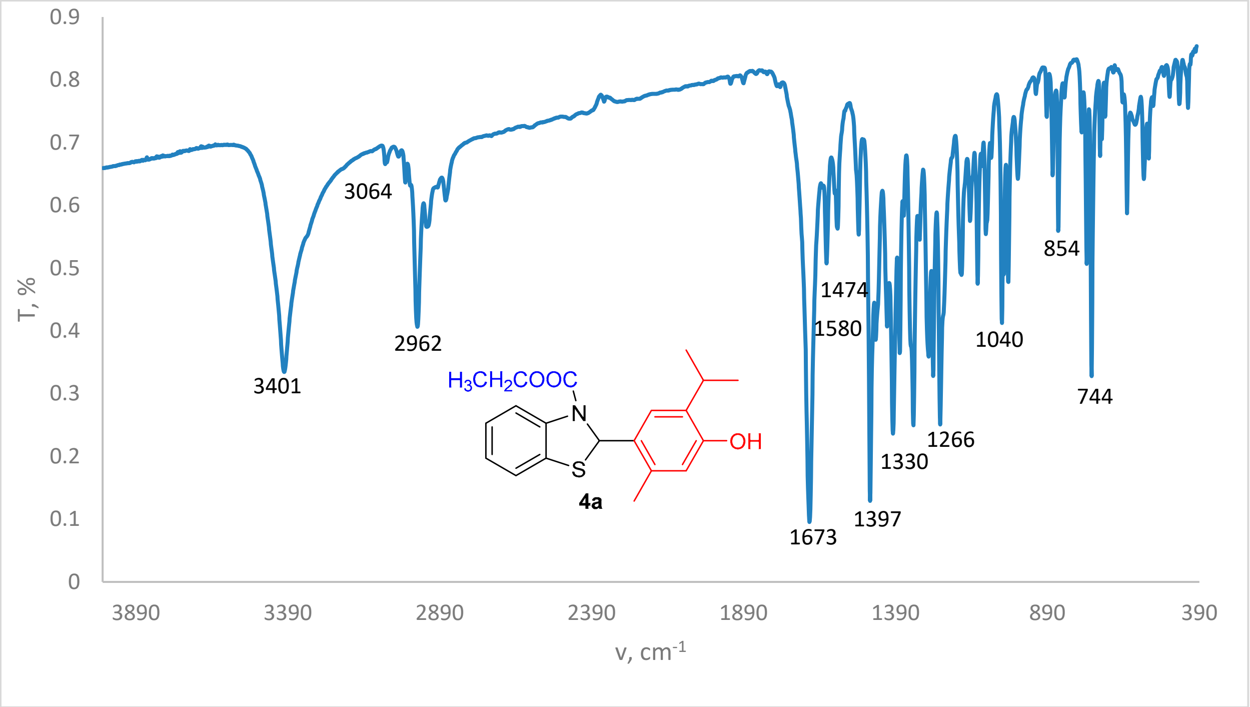

Figure S4 ESI-HRMS spectrum of compound **4a**

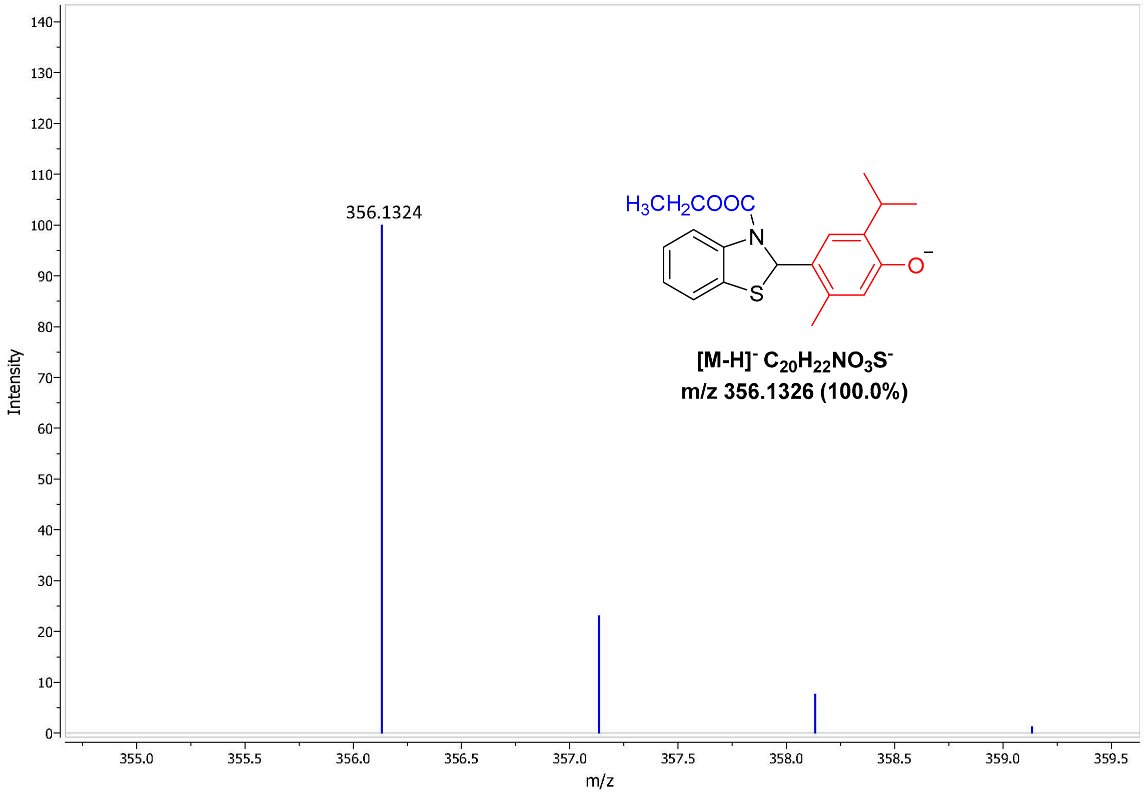

Figure S5 ^{1}H-NMR spectrum of **4b** - DMSO-d6, 80 °C, 600 MHz

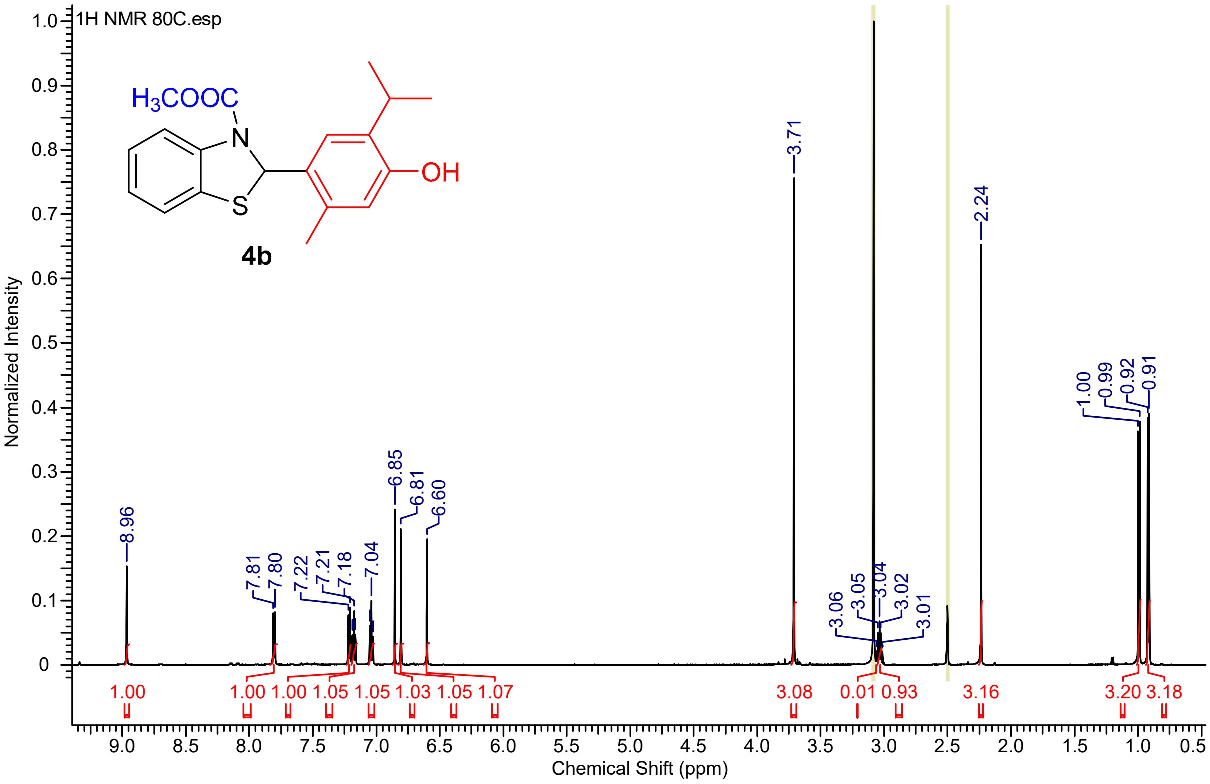

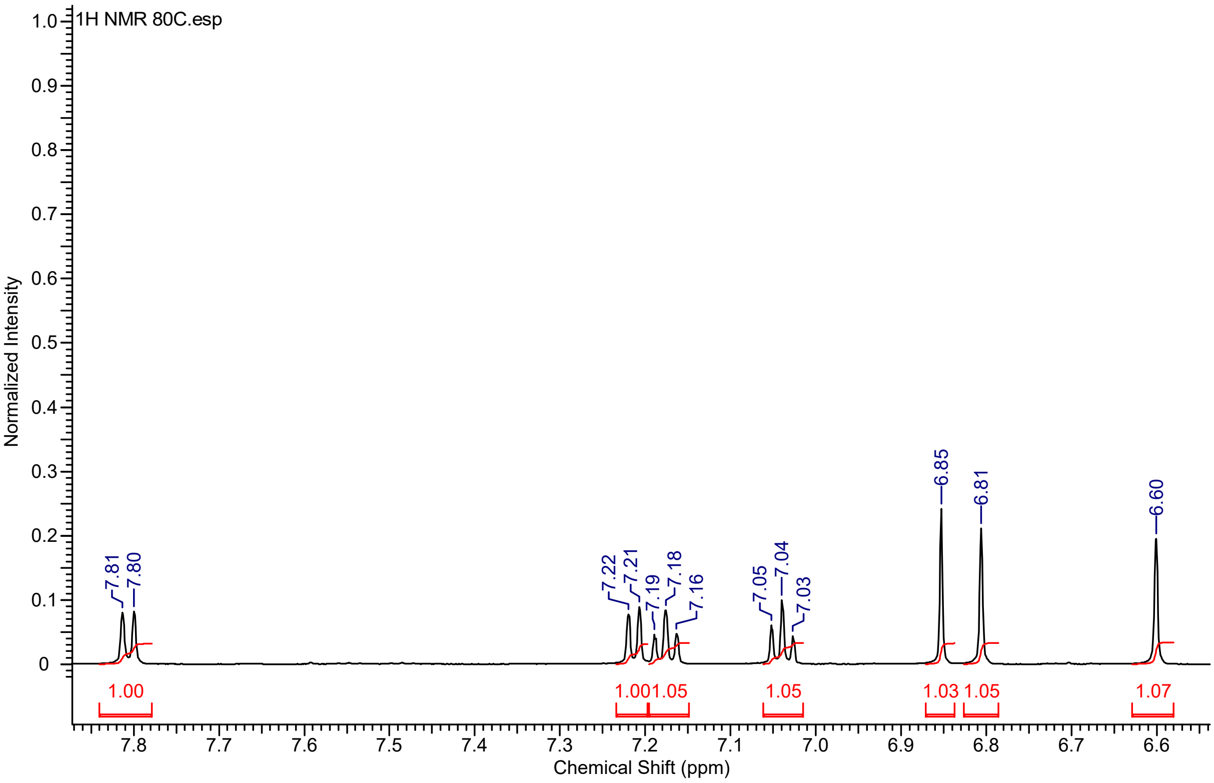

Figure S6 ^{13}C-NMR spectrum of **4b -** DMSO-d$_6$, 80 °C, 150 MHz

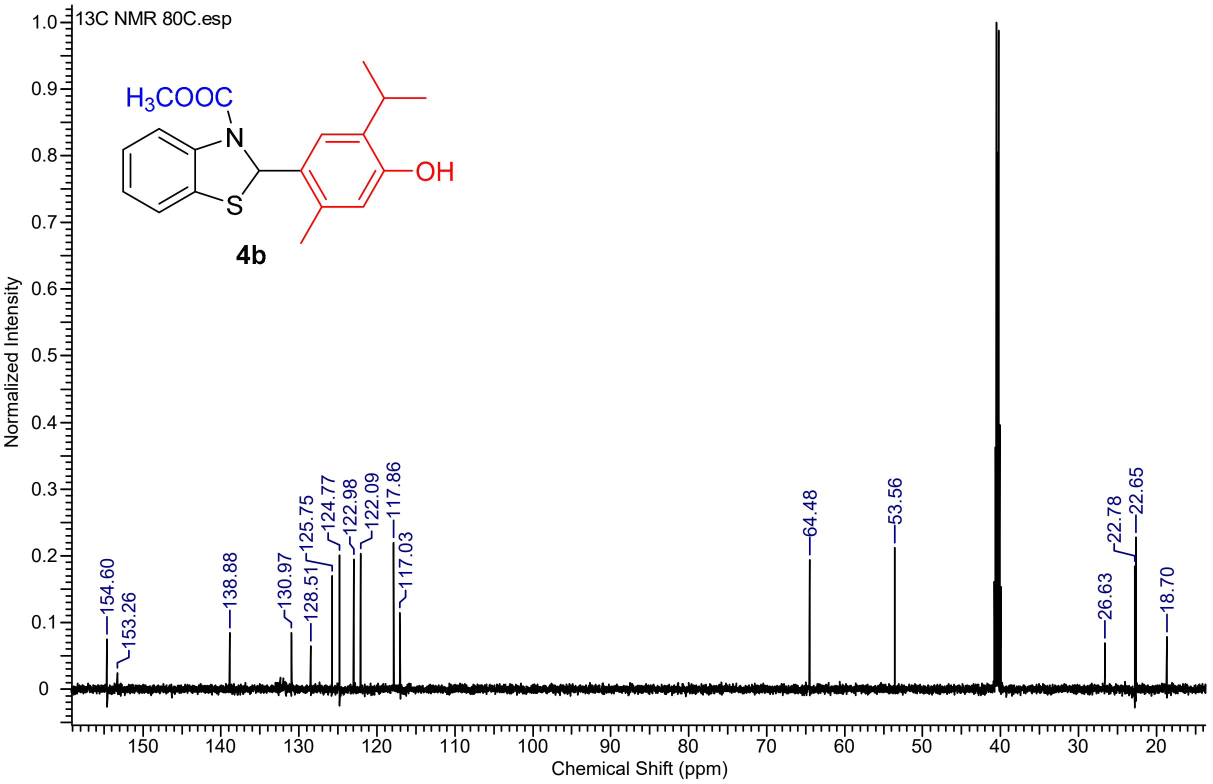

Figure S7 FT-IR spectrum of compound **4b**, KBr tablet, cm^{-1}

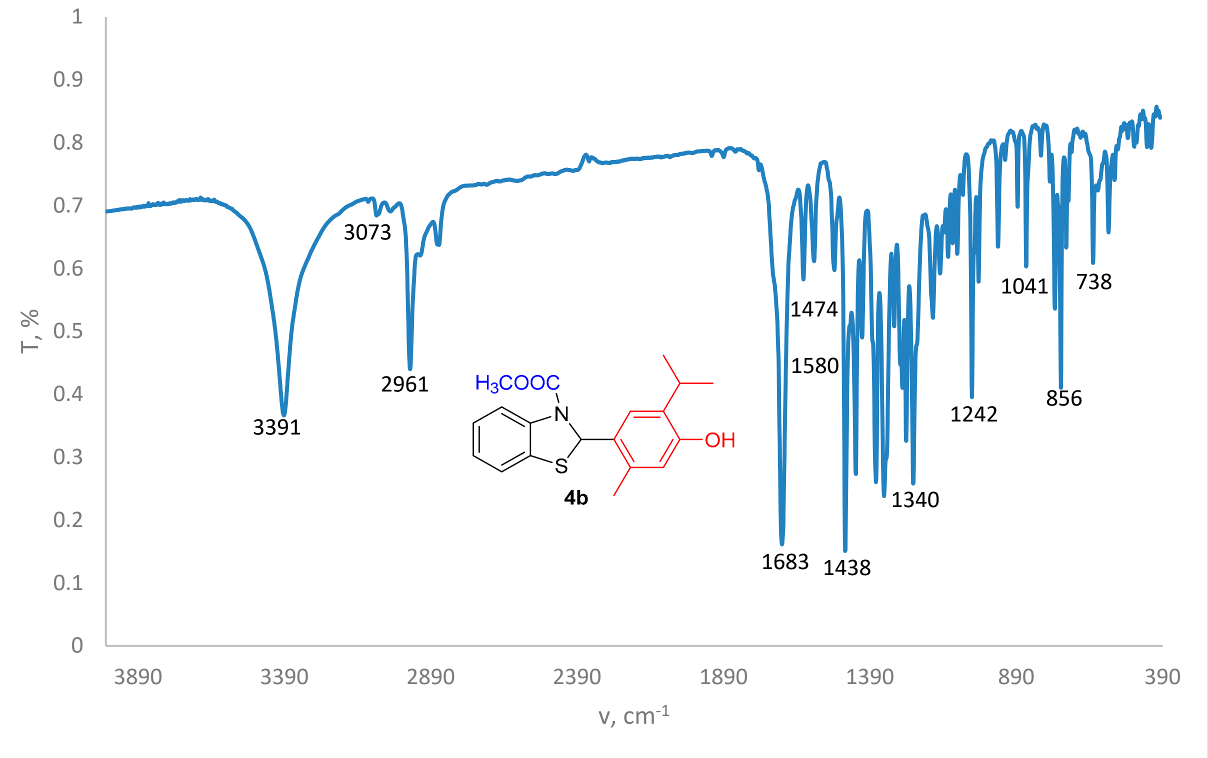

Figure S8 ESI-HRMS spectrum of compound **4b**

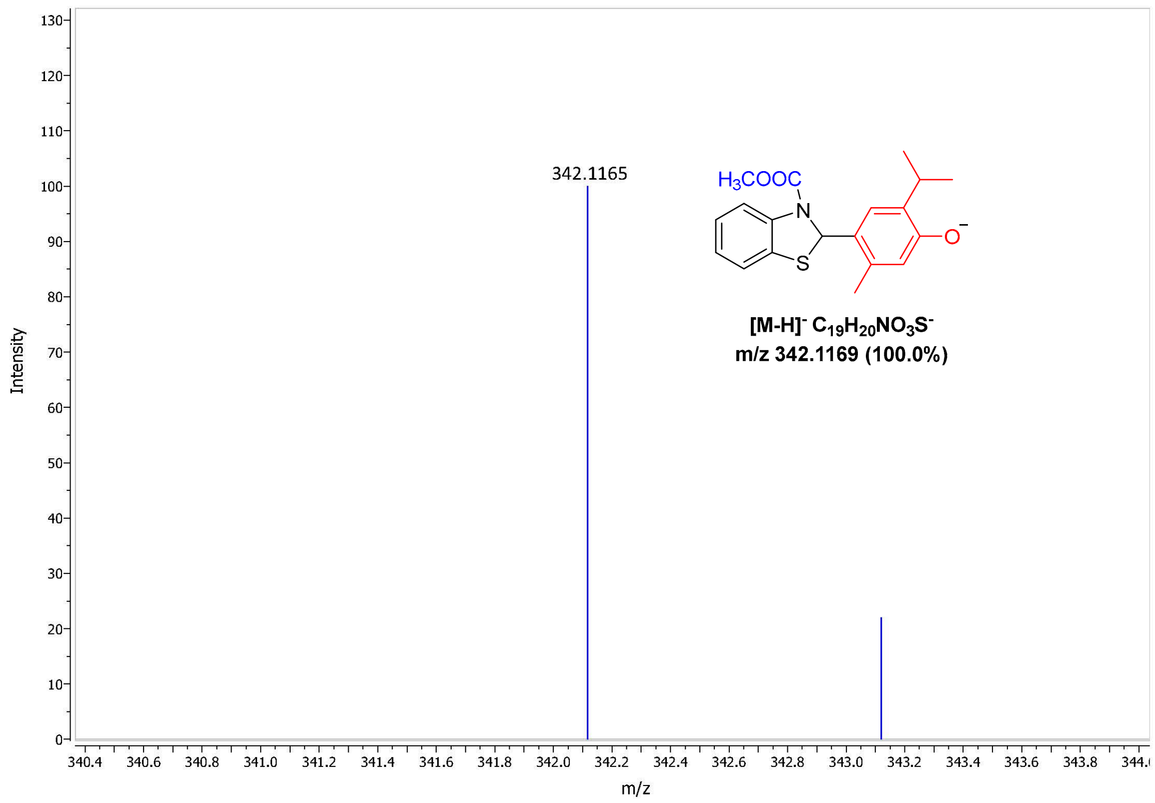

Figure S9 ^{1}H-NMR spectrum of **4c** - DMSO-d$_6$, 80 ºC, 600 MHz

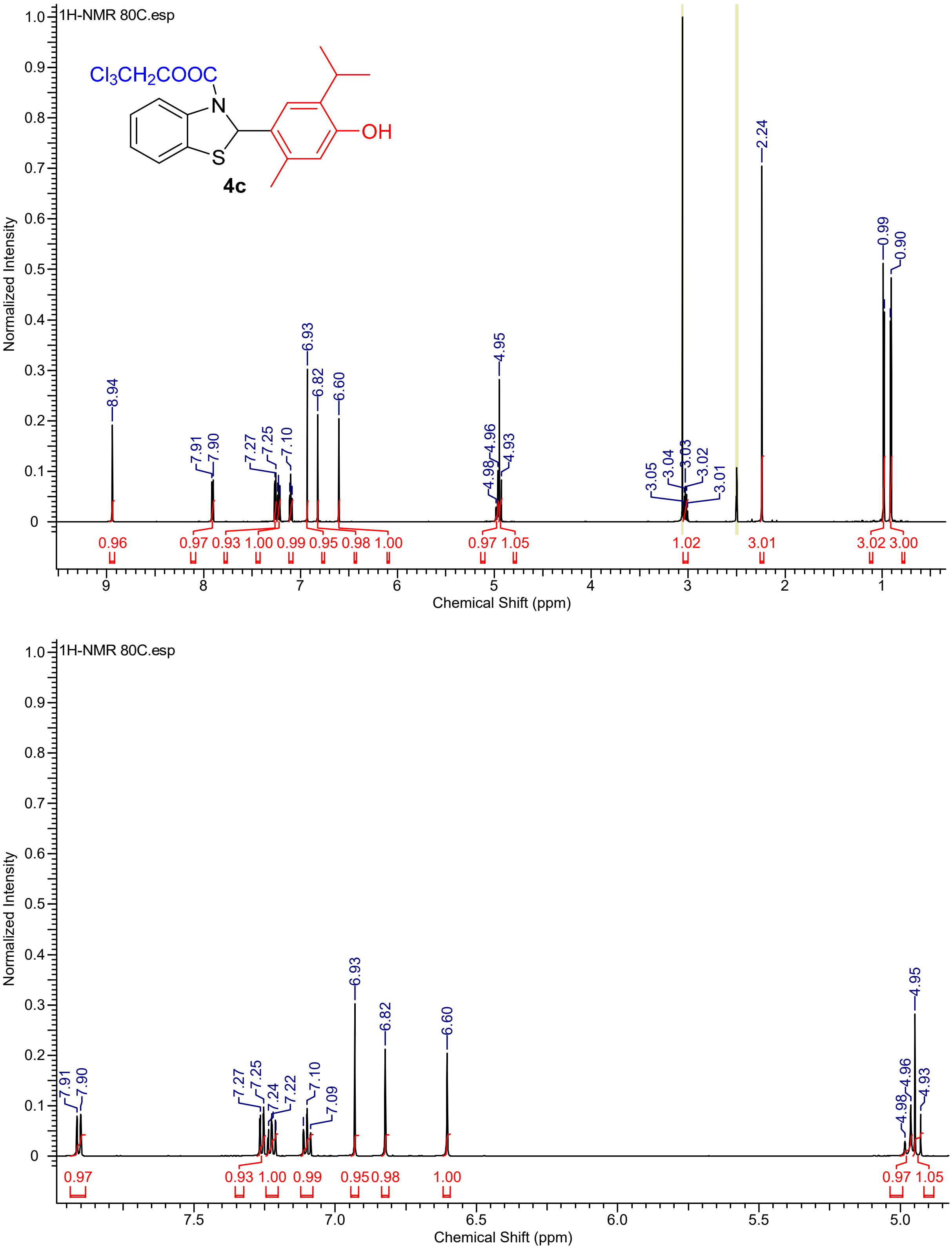

Figure S10 ^{13}C-NMR spectrum of **4c** - DMSO-d$_6$, 80 ºC, 150 MHz

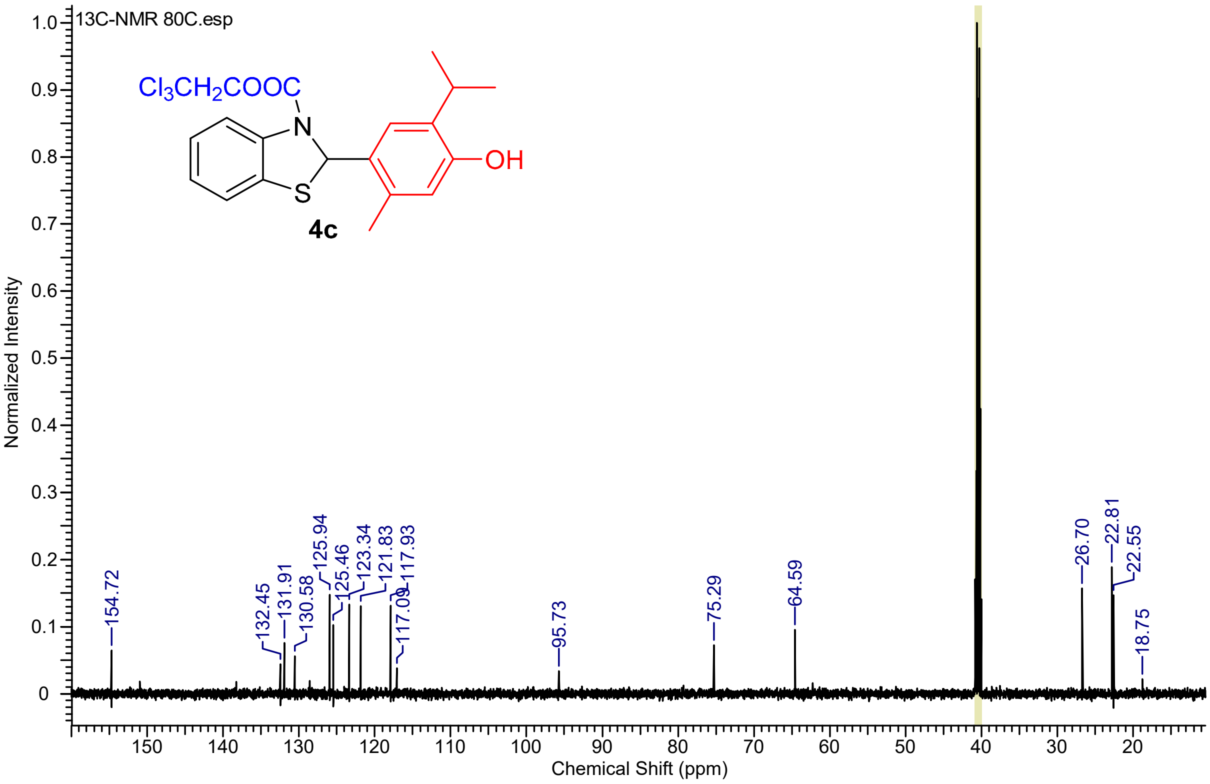

Figure S11 FT-IR spectrum of compound **4c**, KBr tablet, cm^{-1}

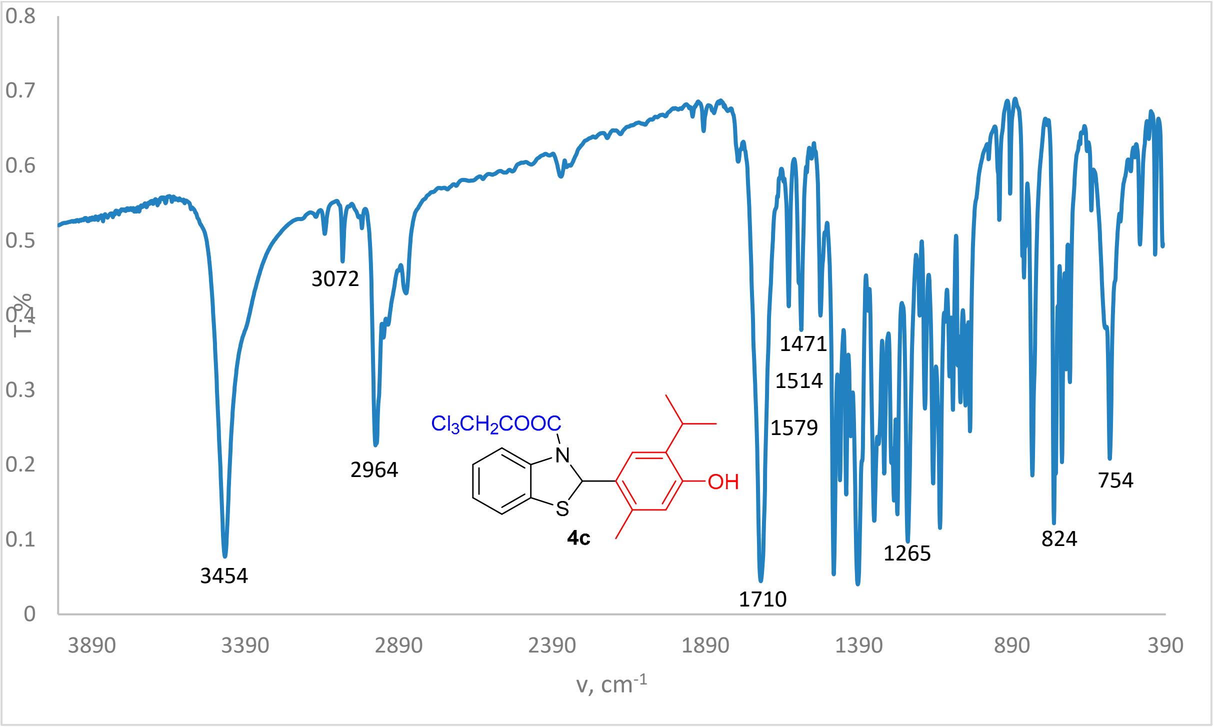

Figure S12 ESI-HRMS spectrum of compound **4c**

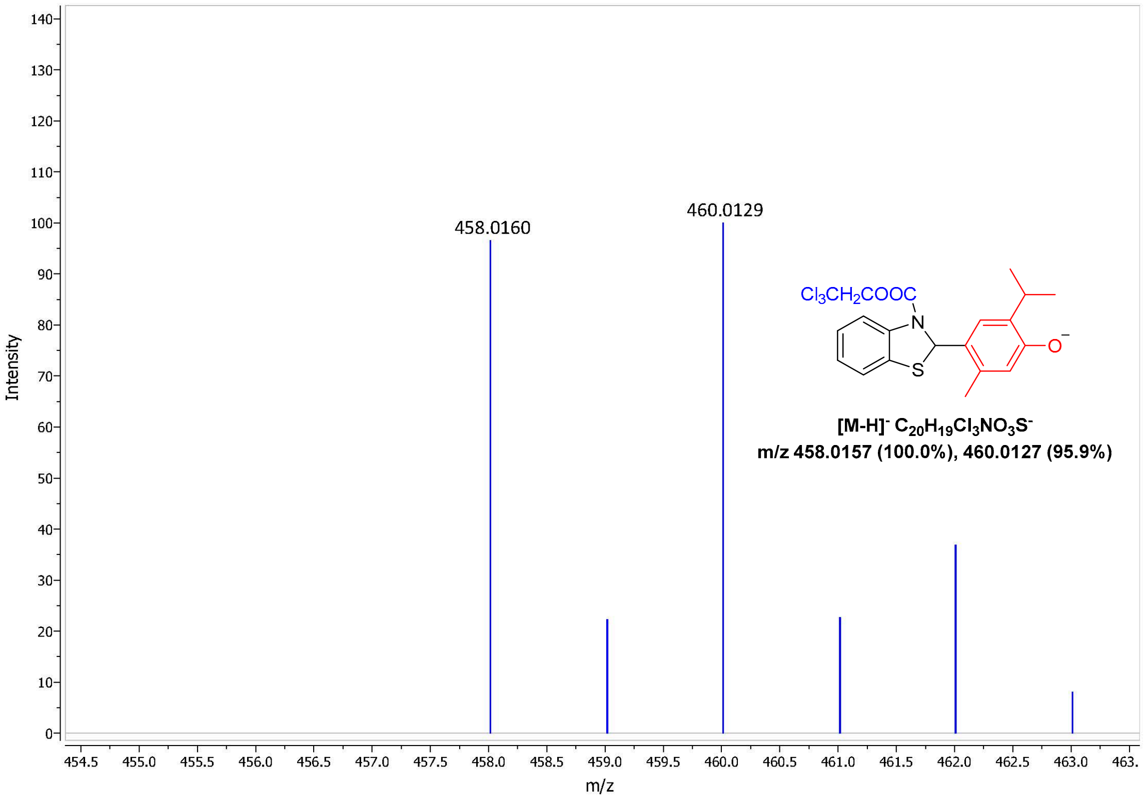

Figure S13 ^{1}H-NMR spectrum of **5a** - DMSO-d6, 80 °C, 600 MHz

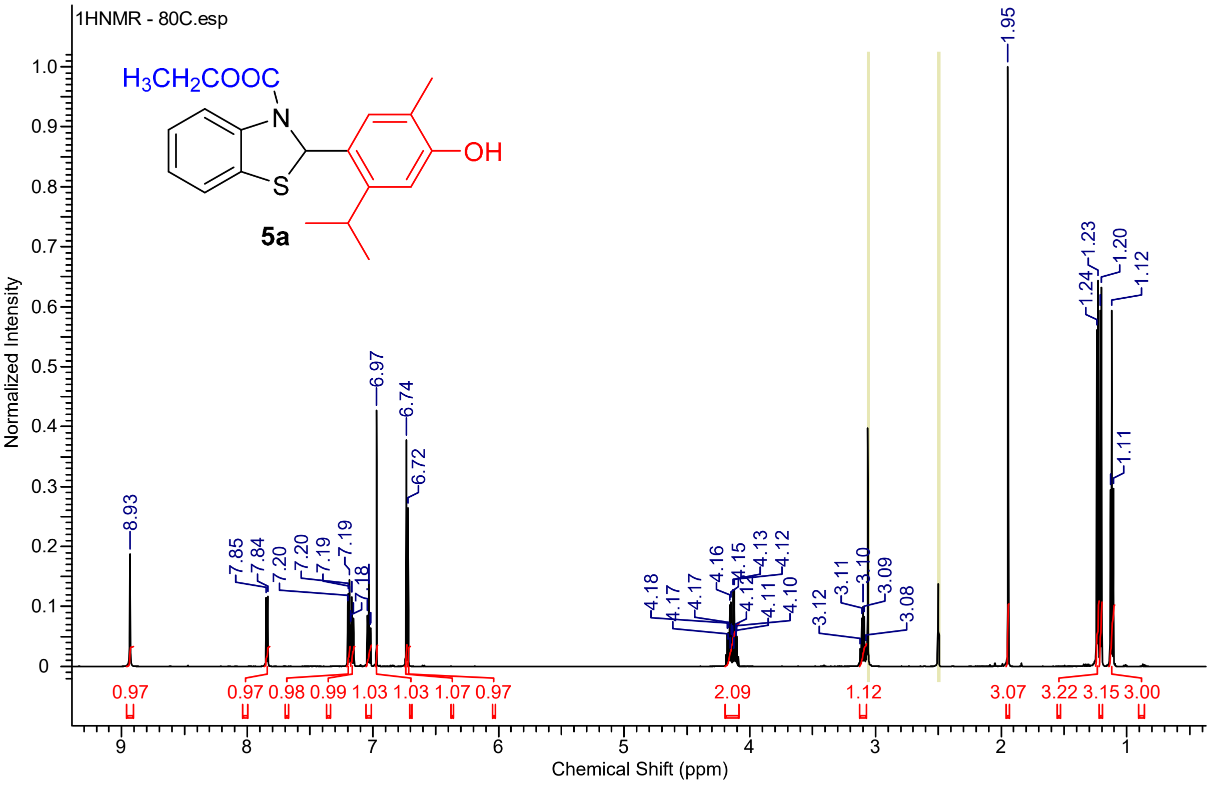

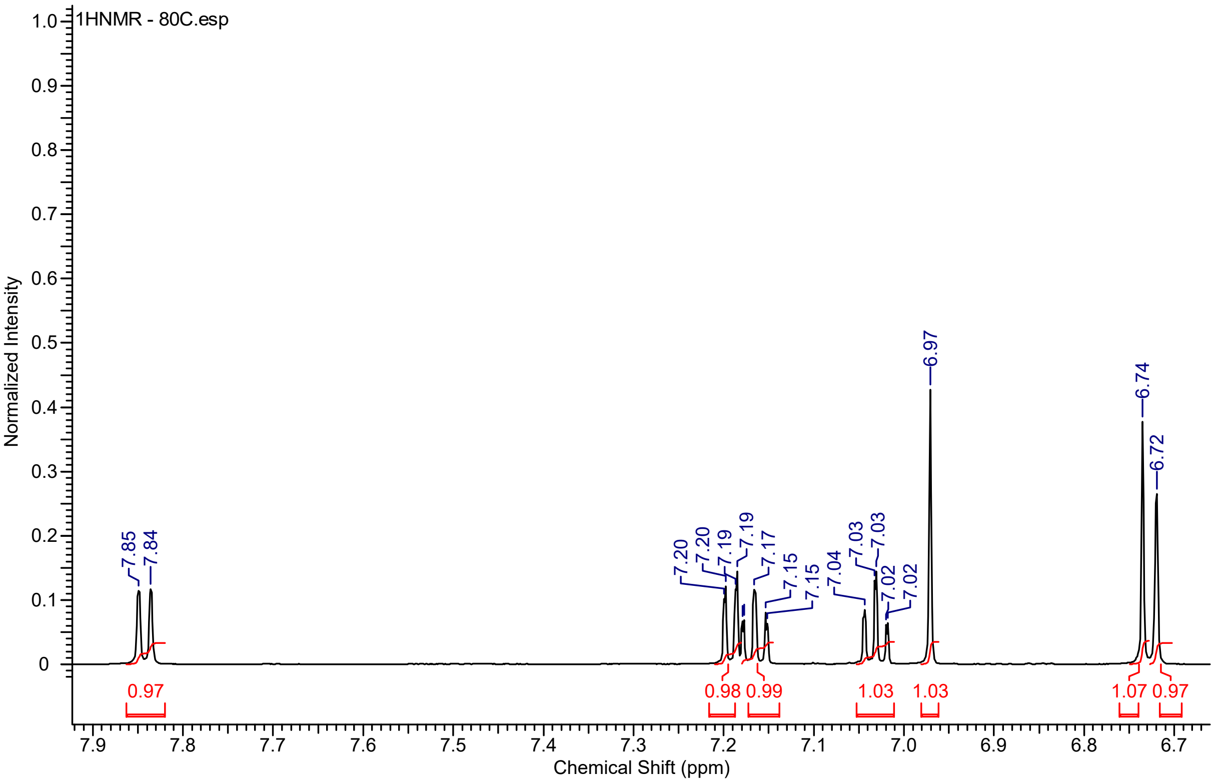

Figure S14 ^{13}C-NMR spectrum of **5a -** DMSO-d6, 80 ºC, 150 MHz

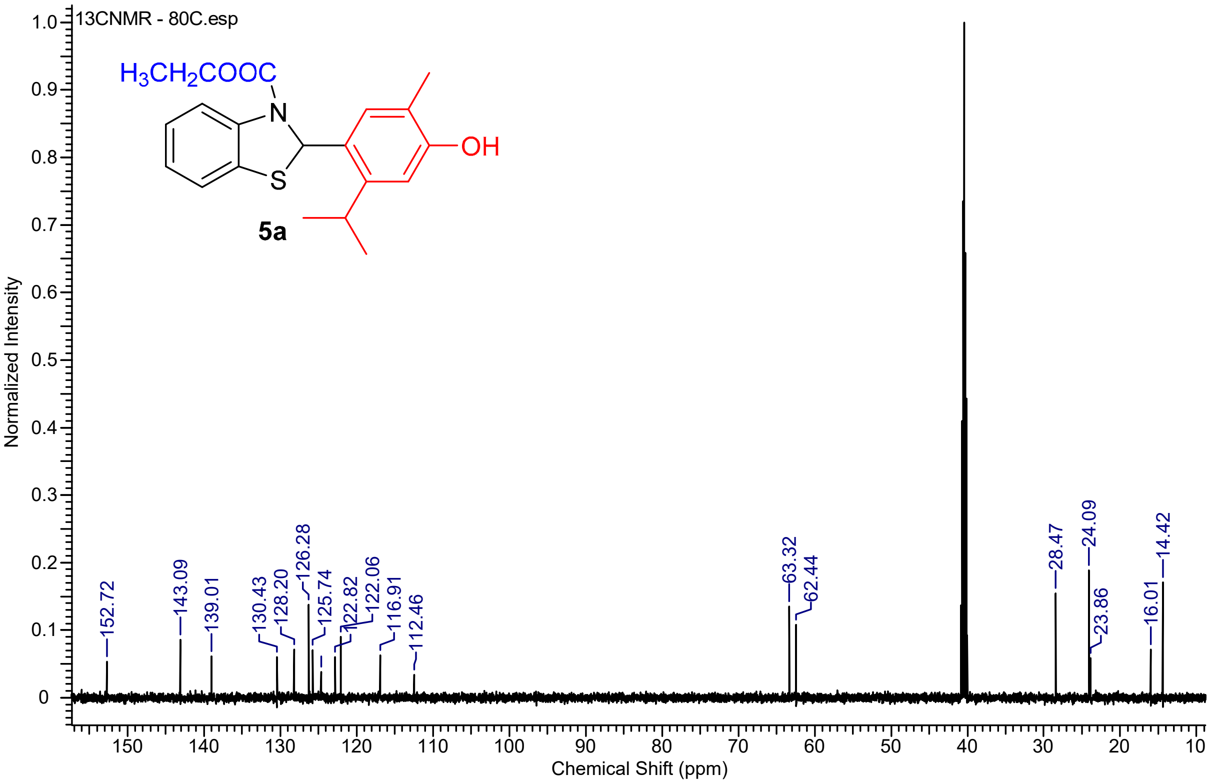

Figure S15 FT-IR spectrum of compound **5a**, KBr tablet, cm^{-1}

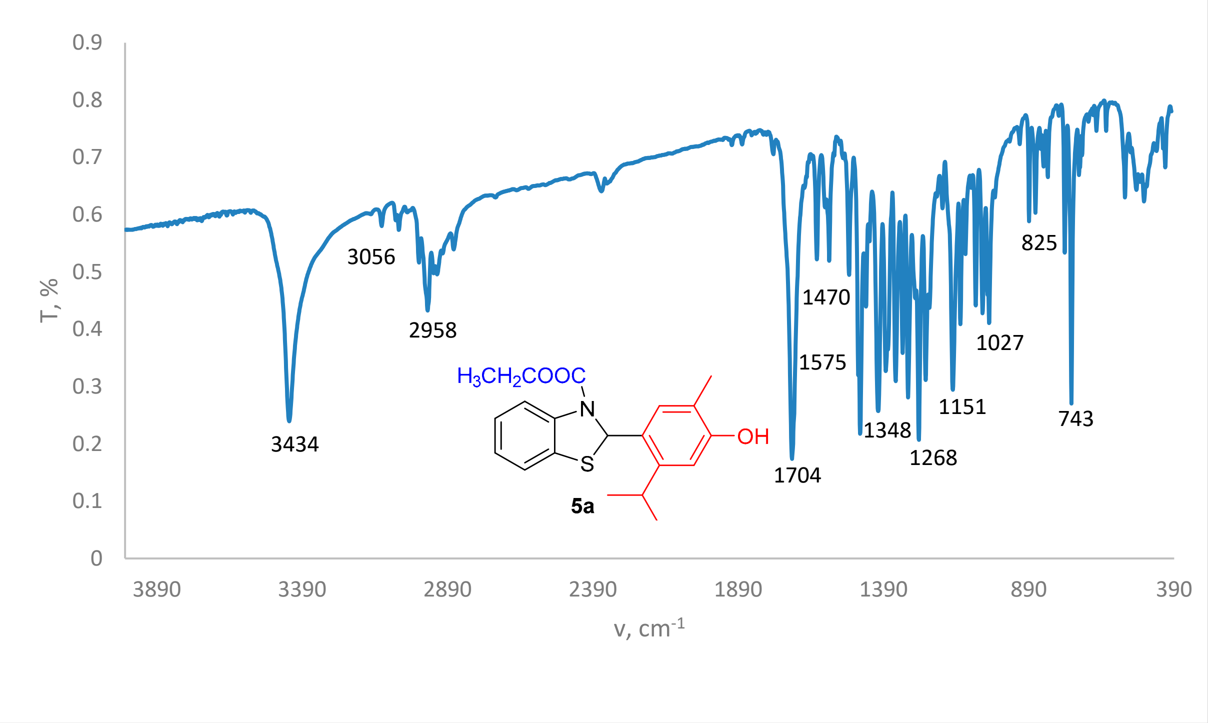

Figure S16 ESI-HRMS spectrum of compound **5a**

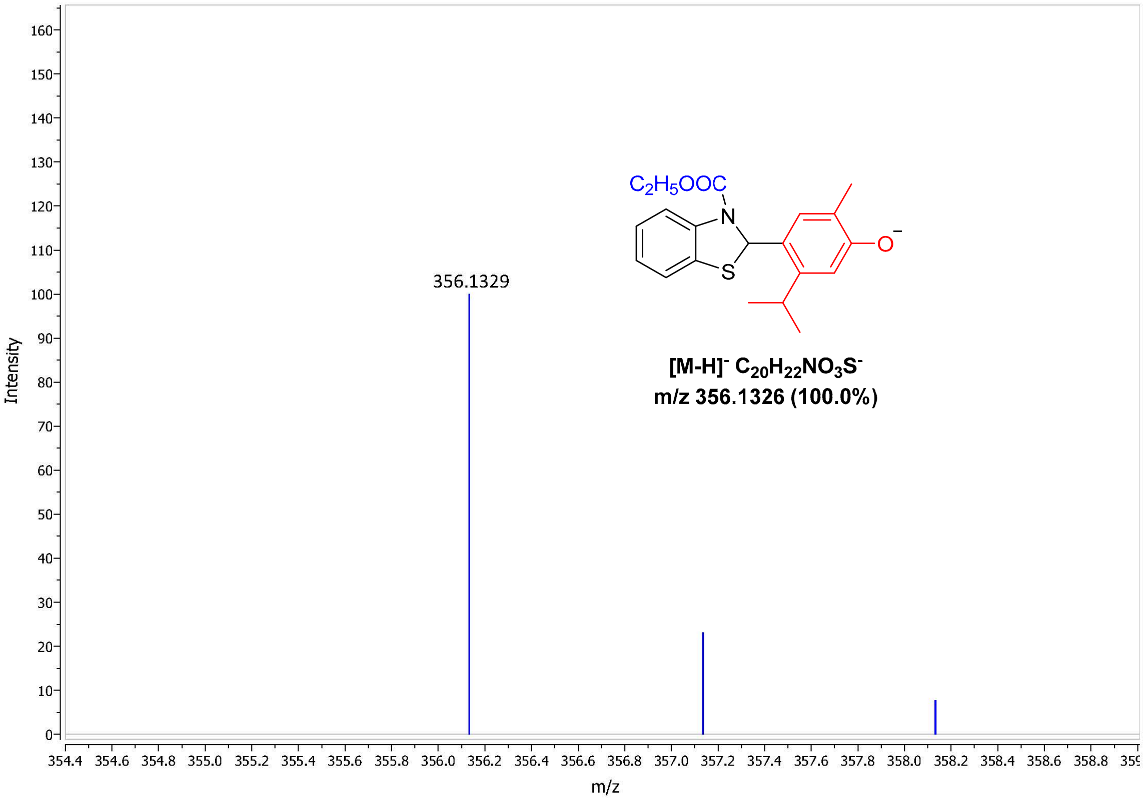

Figure S17 ^{1}H-NMR spectrum of **5b** - DMSO-d$_6$, 80 °C, 600 MHz

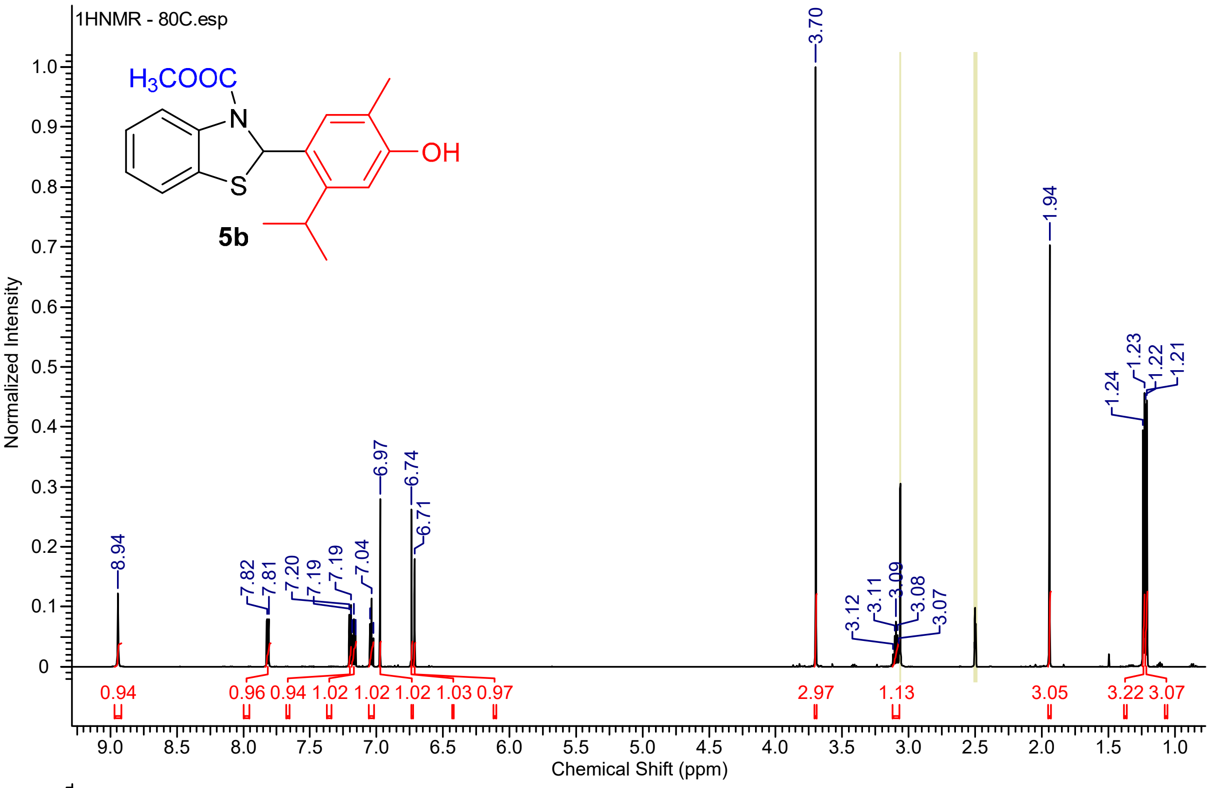

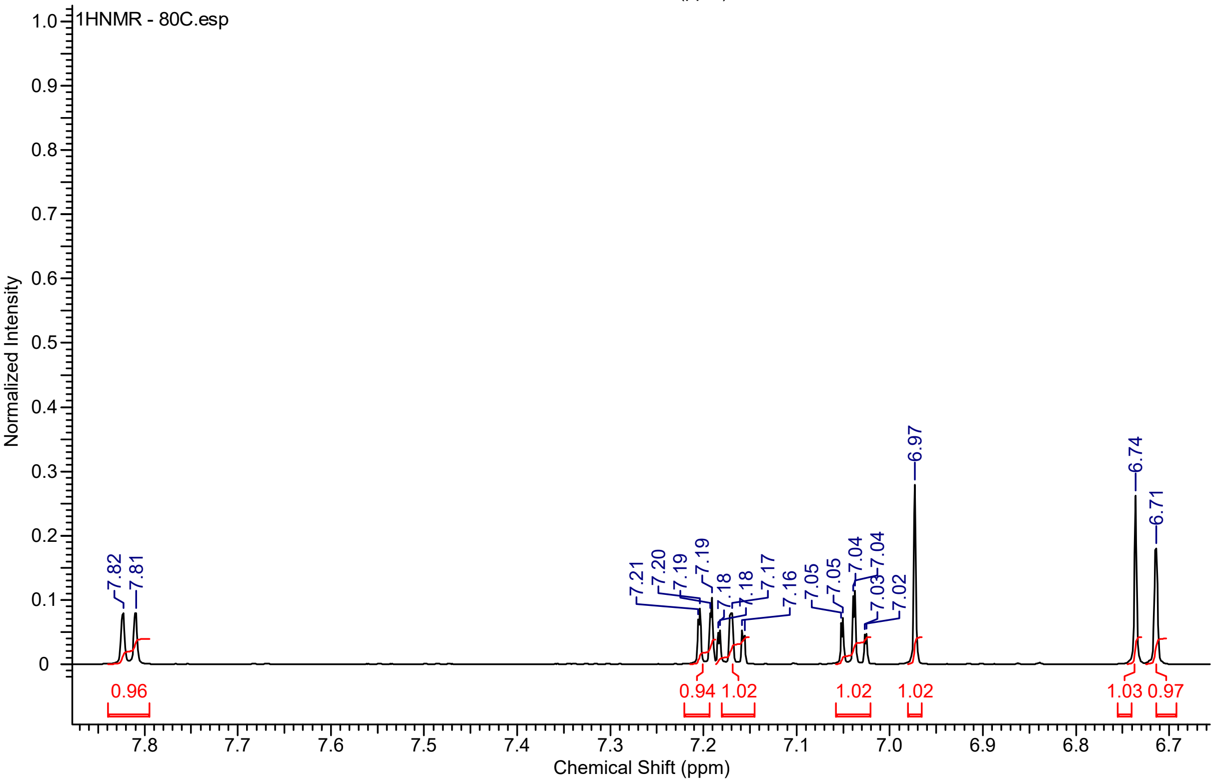

Figure S18 ^{13}C-NMR spectrum of **5b** - DMSO-d₆, 80 ºC, 150 MHz

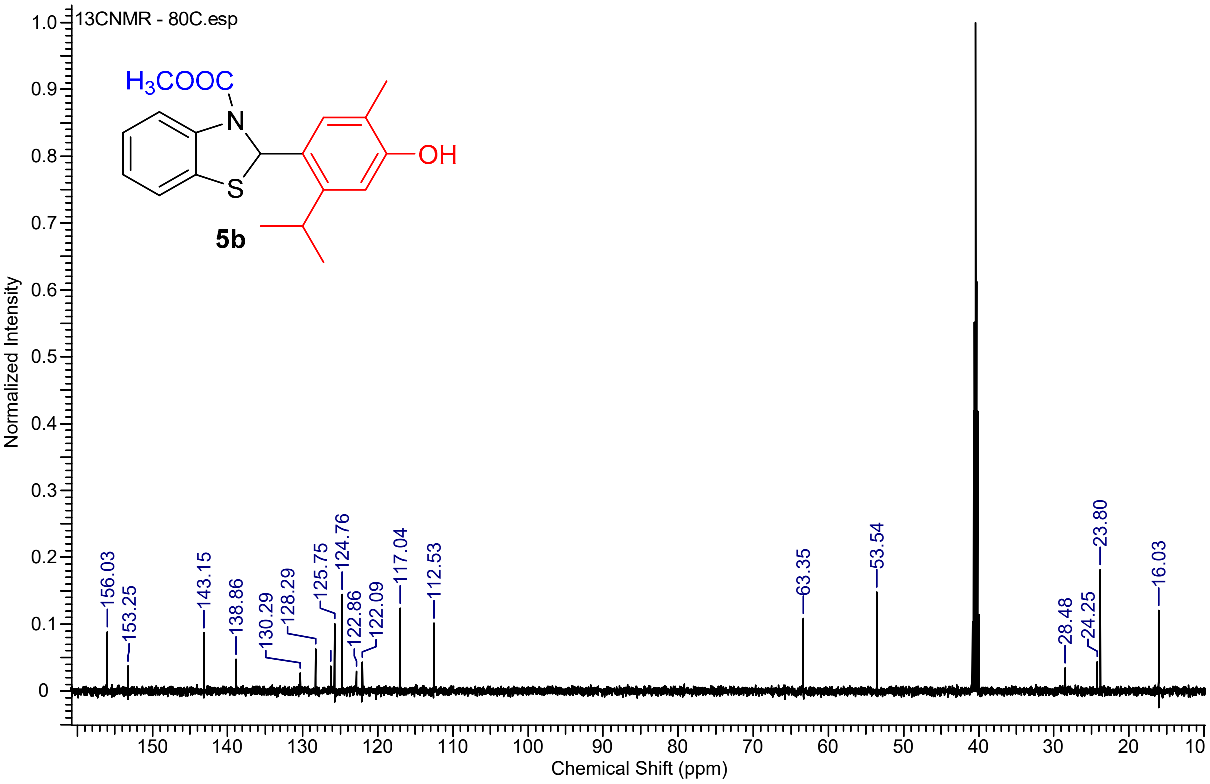

Figure S19 FT-IR spectrum of compound **5b**, KBr tablet, cm^{-1}

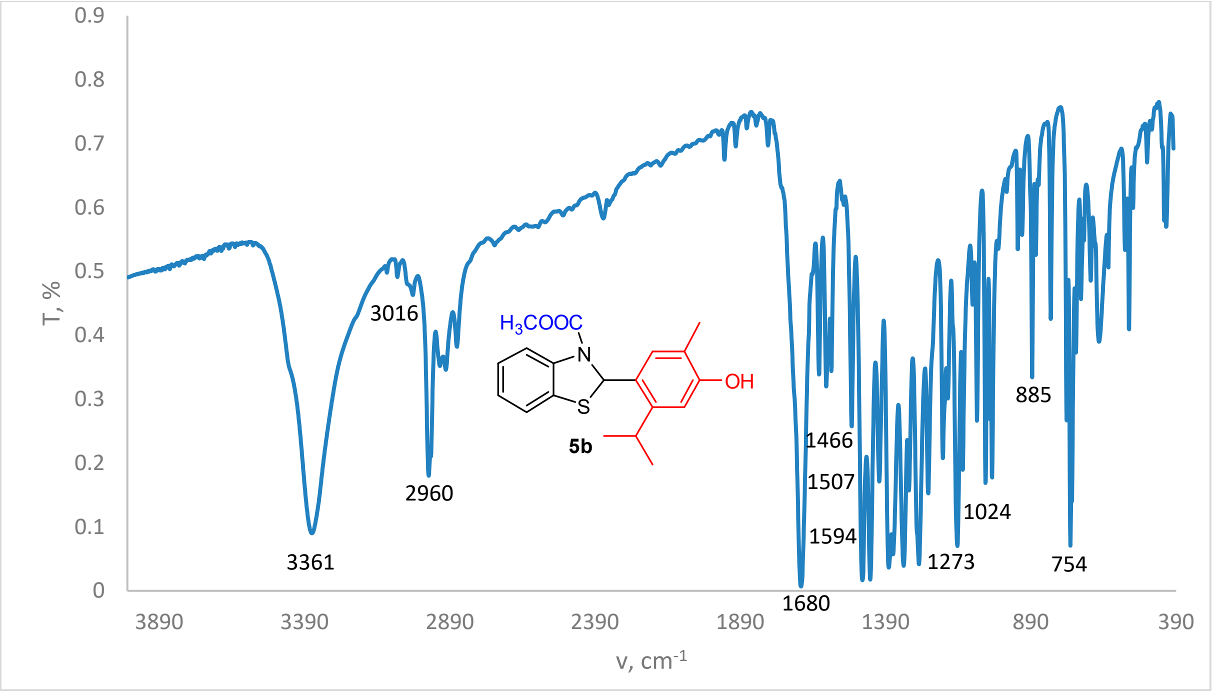

Figure S20 ESI-HRMS spectrum of compound **5b**

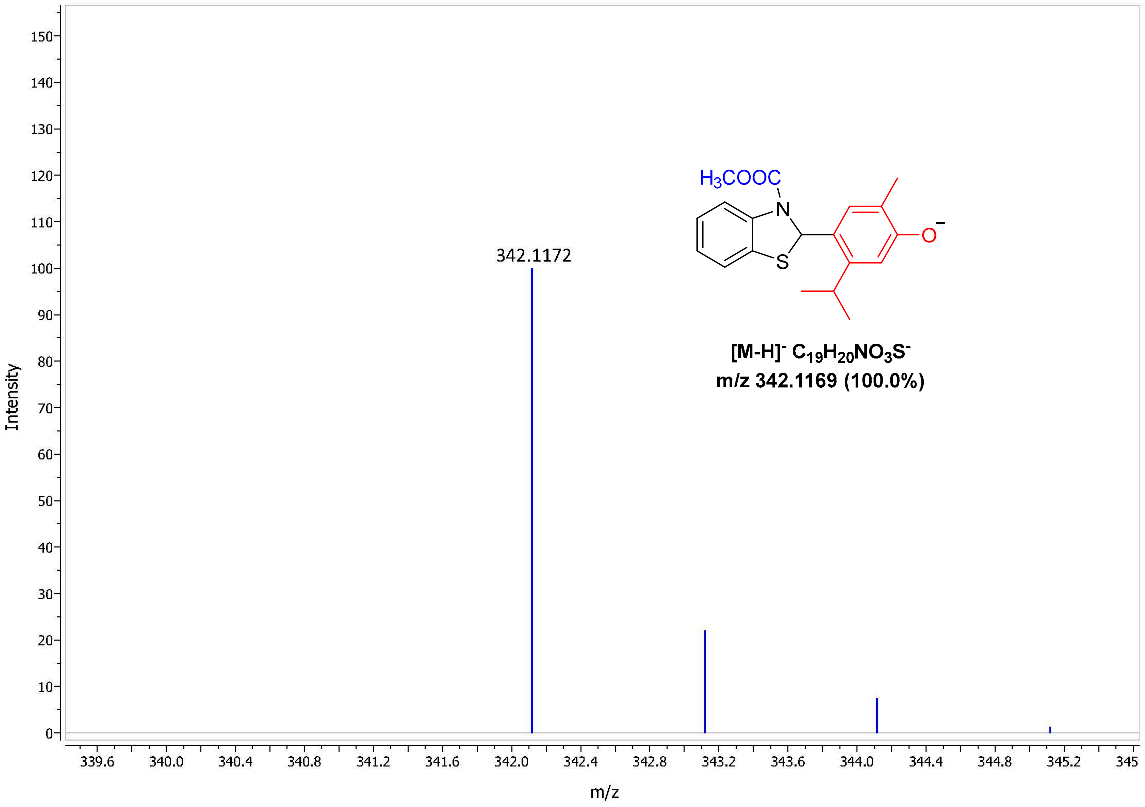

Figure S21 ^{1}H-NMR spectrum of **5c** - DMSO-d6, 80 °C, 600 MHz

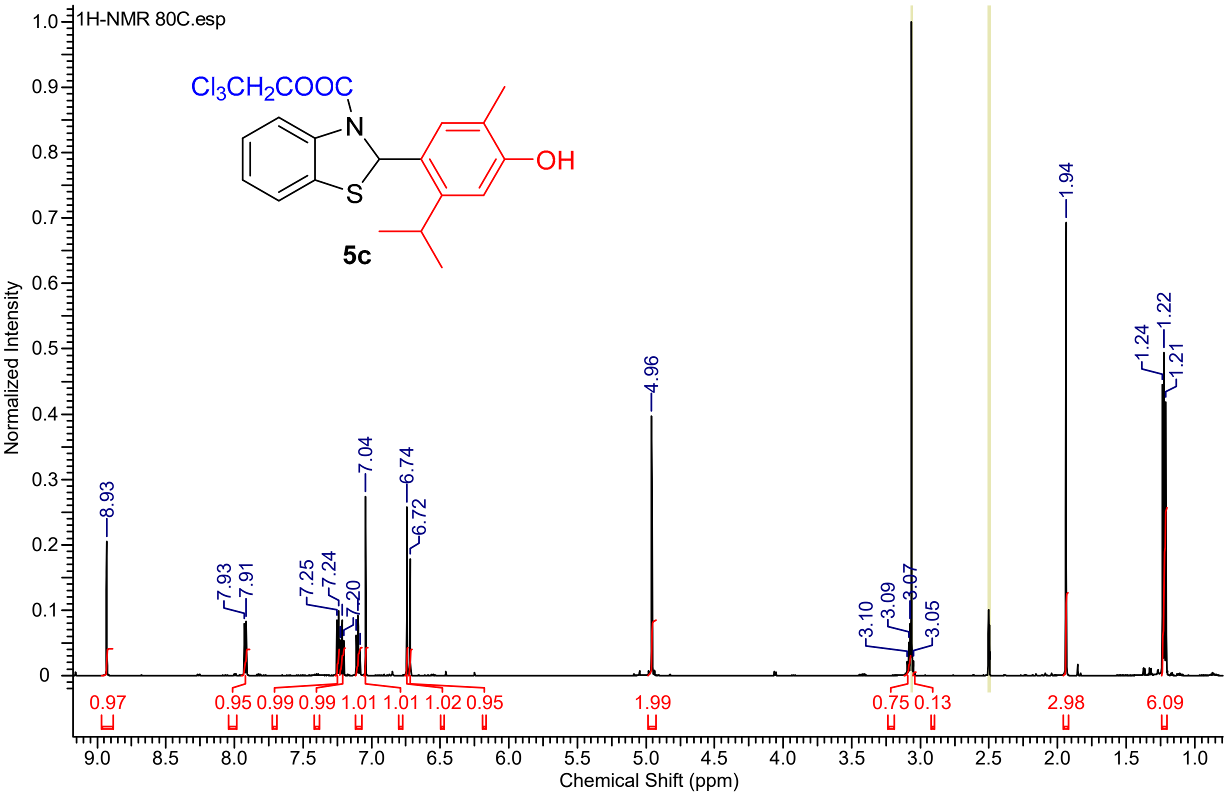

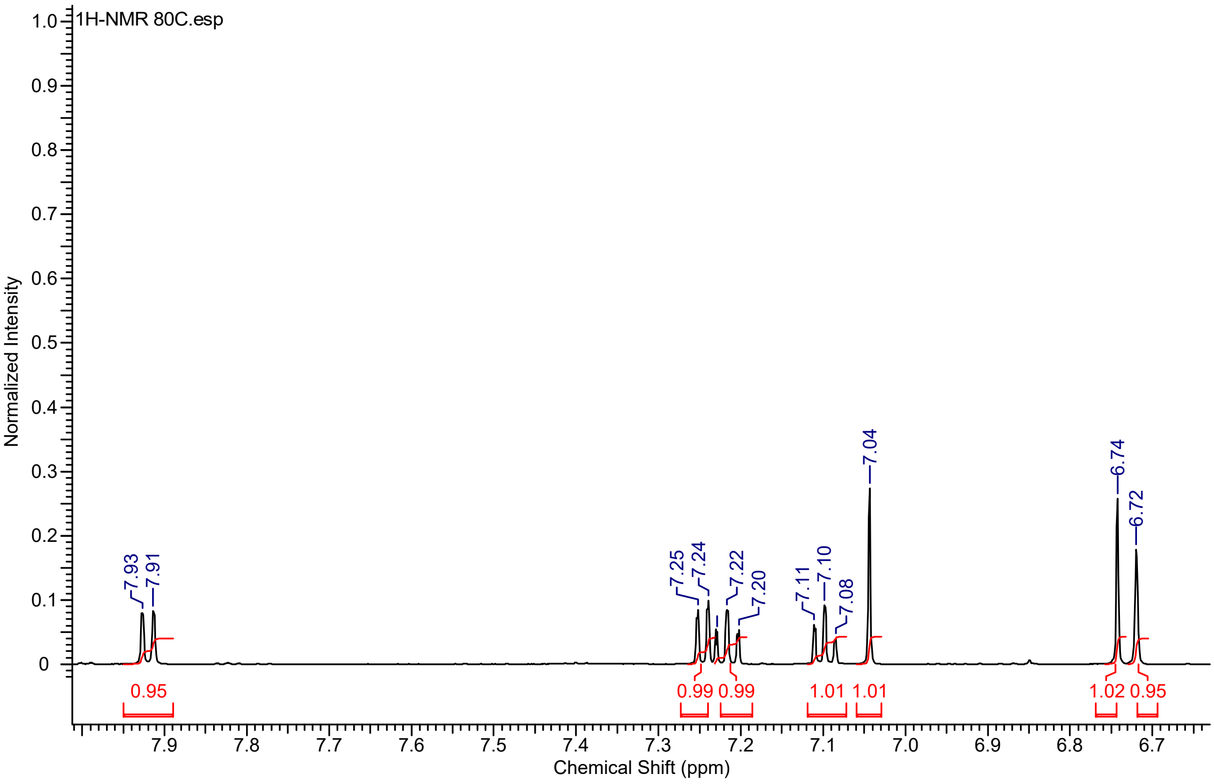

Figure S22 ^{13}C-NMR spectrum of **5c** - DMSO-d6, 80 ºC, 150 MHz

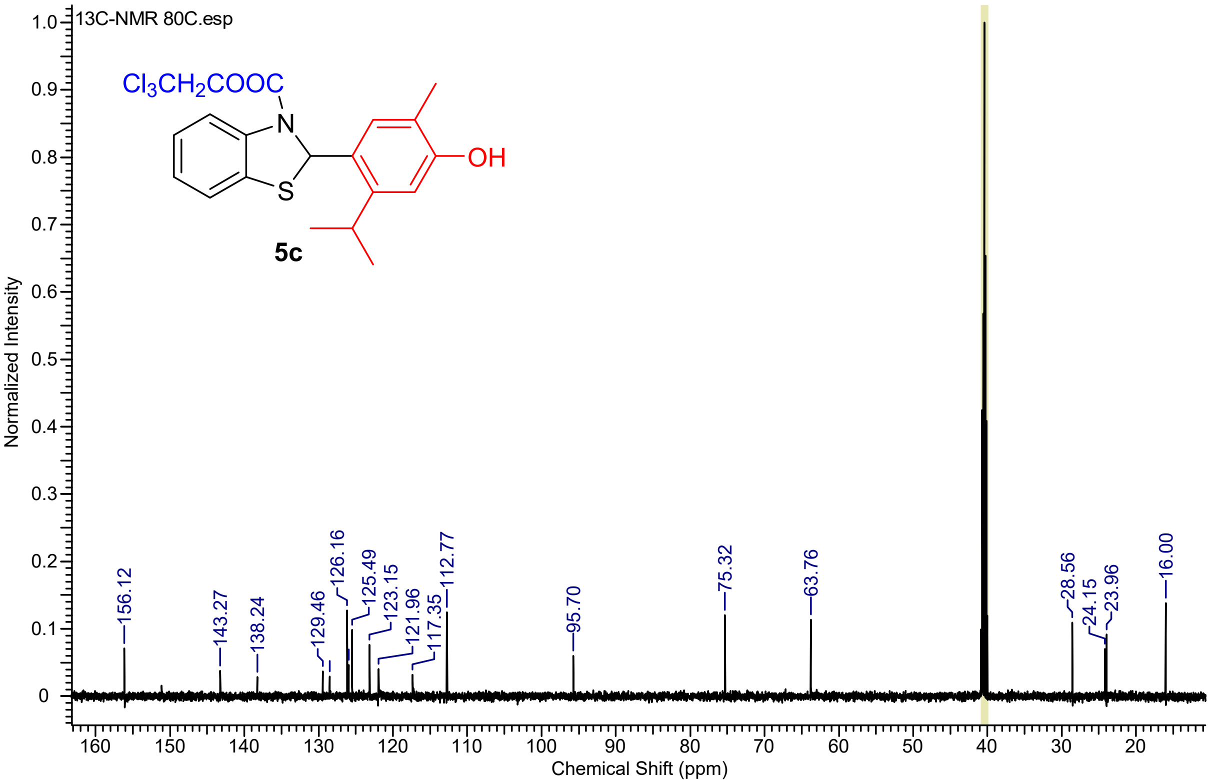

Figure S23 FT-IR spectrum of compound **5c**, KBr tablet, cm^{-1}

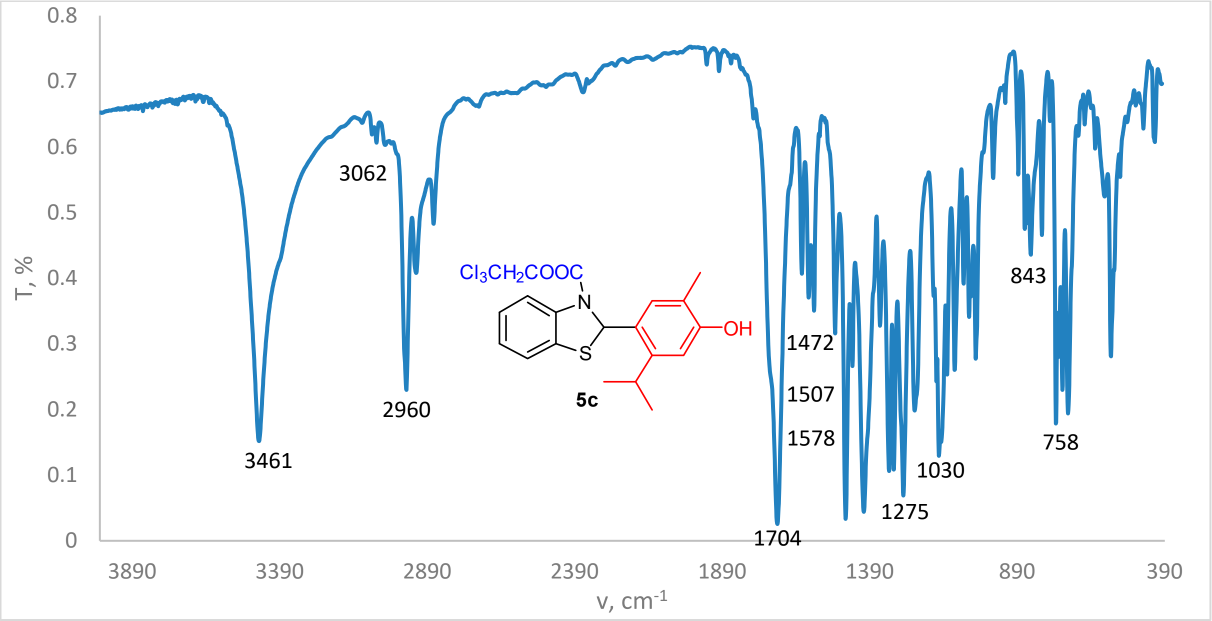

Figure S24 HRMS spectrum of compound **5c**

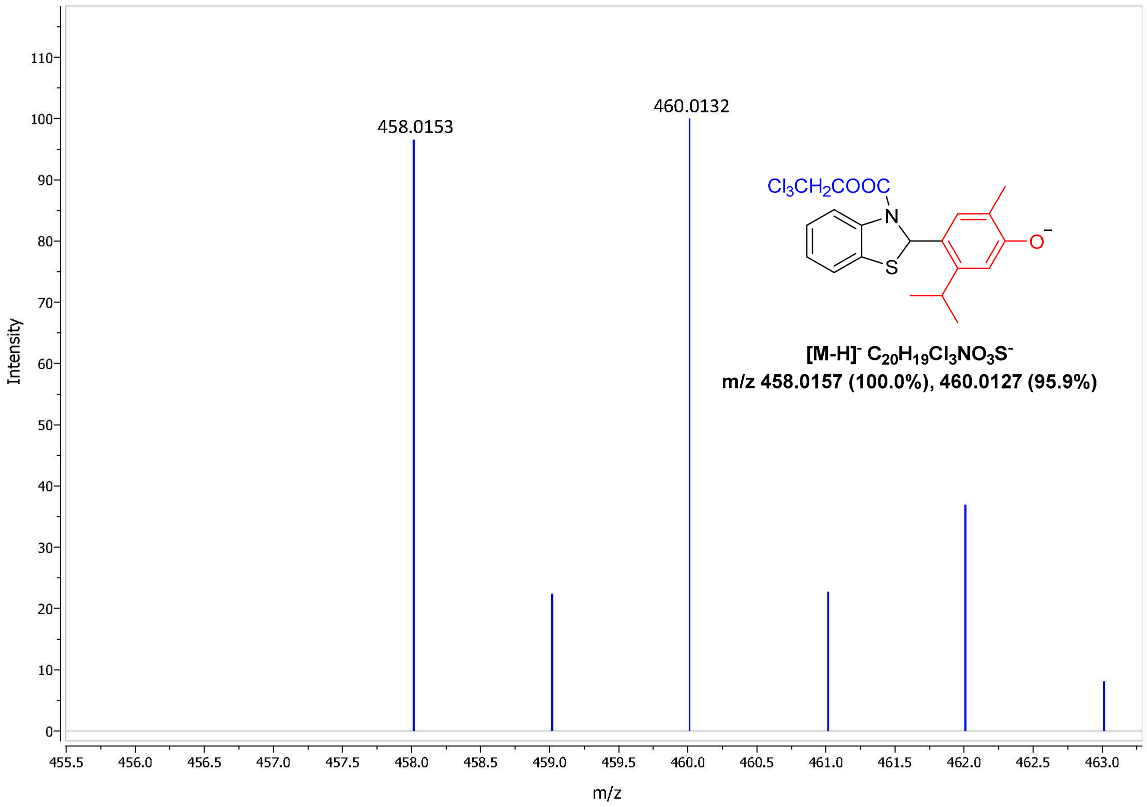

Figure S25 ^{1}H-NMR spectrum of **6** - DMSO-d_6, 80 °C, 600 MHz

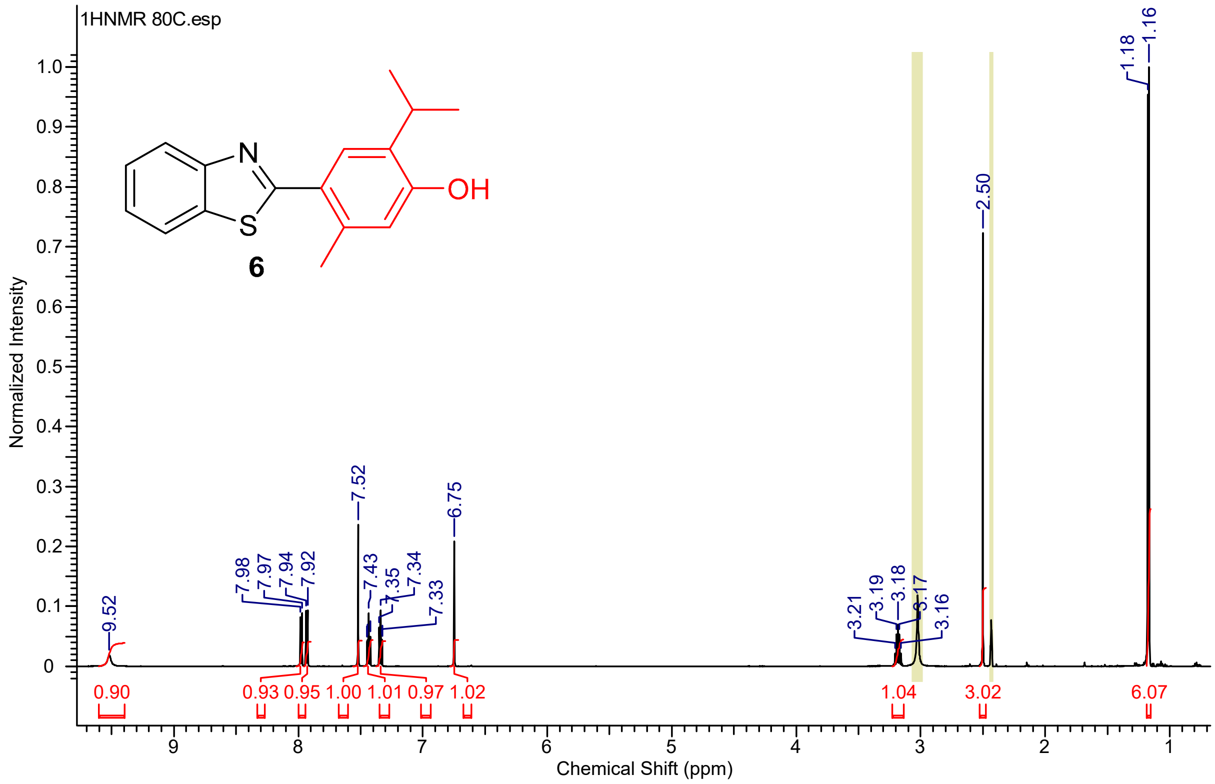

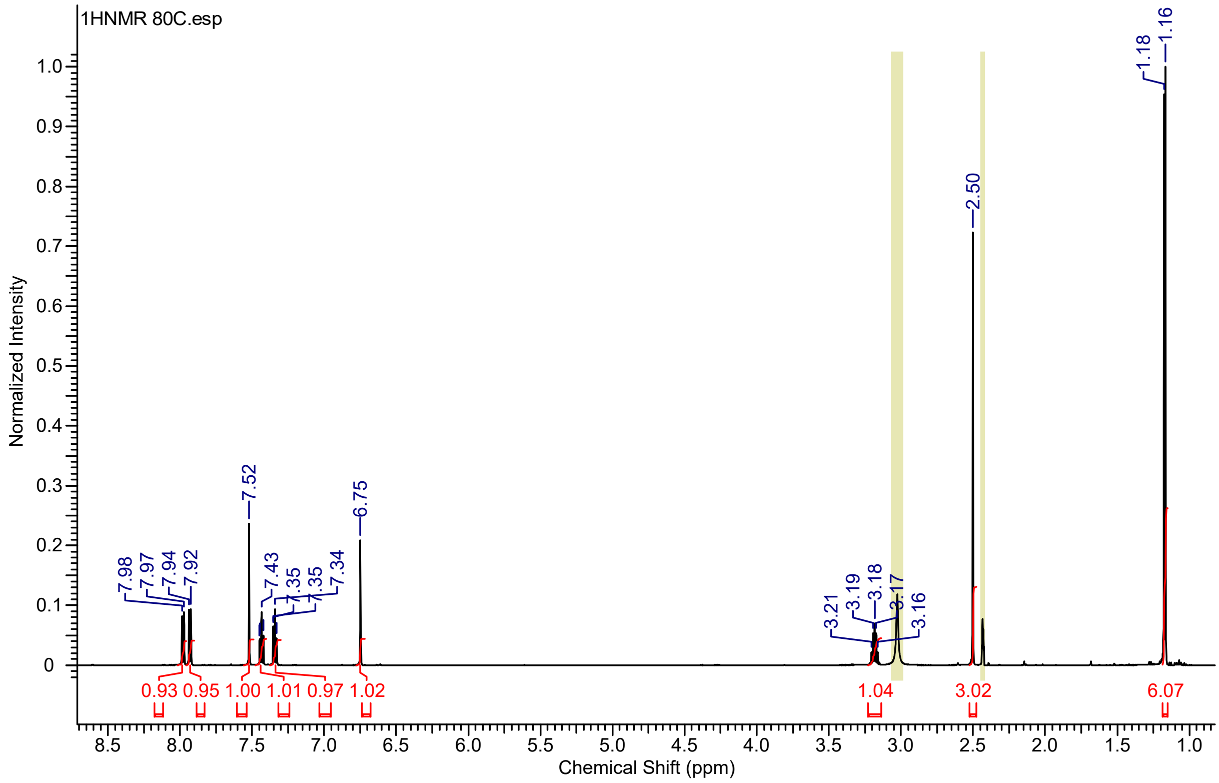

Figure S26 ^{13}C-NMR spectrum of **6** - DMSO-d6, 80 °C, 150 MHz

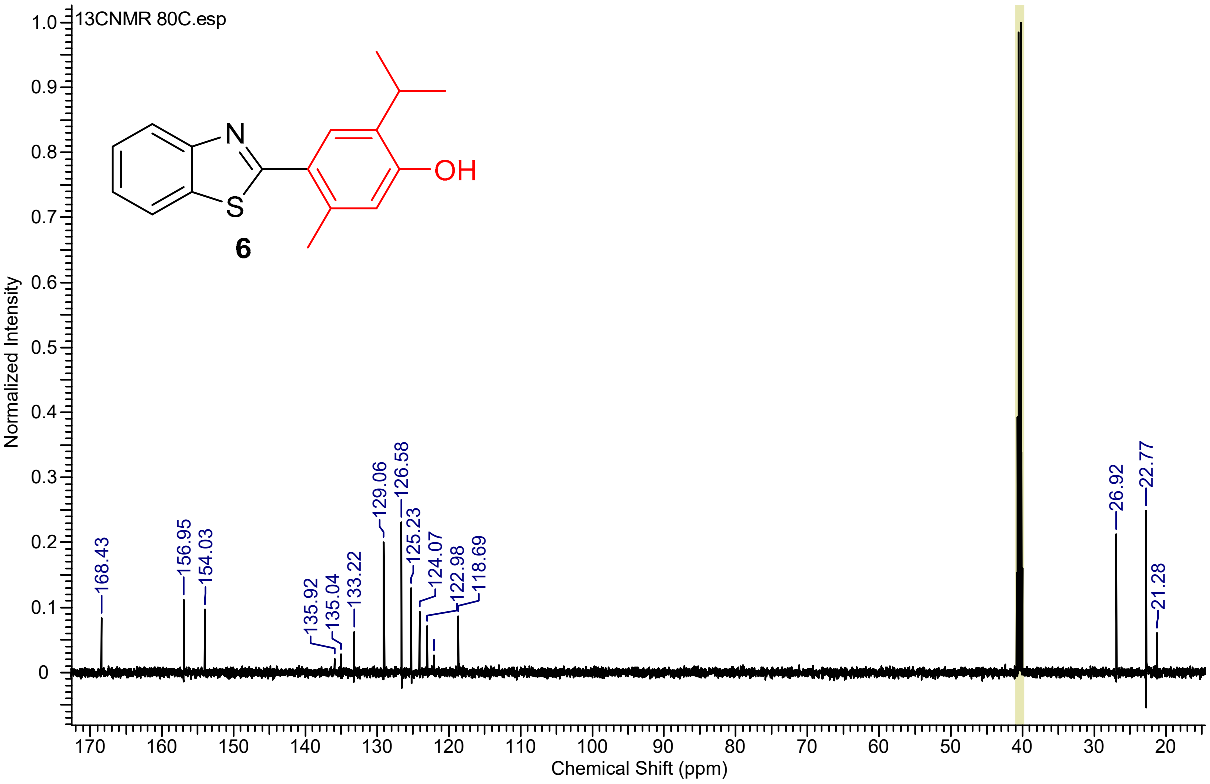

Figure S27 FT-IR spectrum of compound **6**, KBr tablet, cm^{-1}

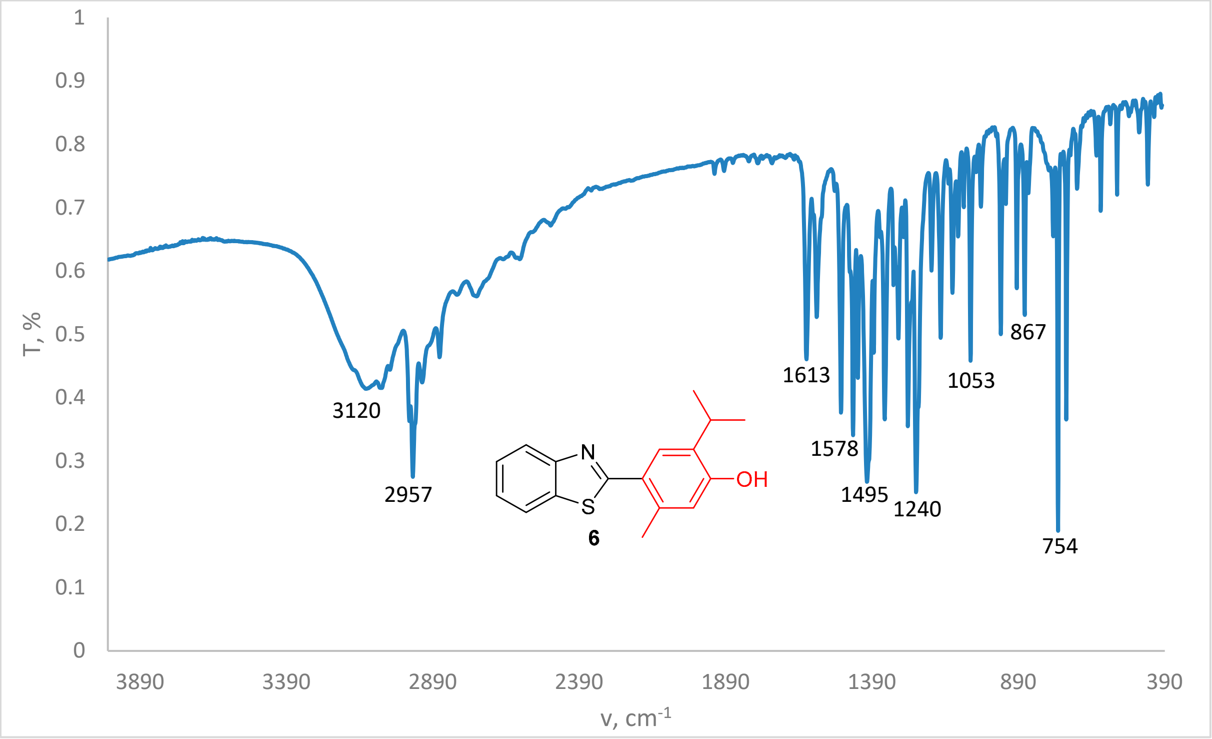

Figure S28 ESI-HRMS spectrum of compound **6**

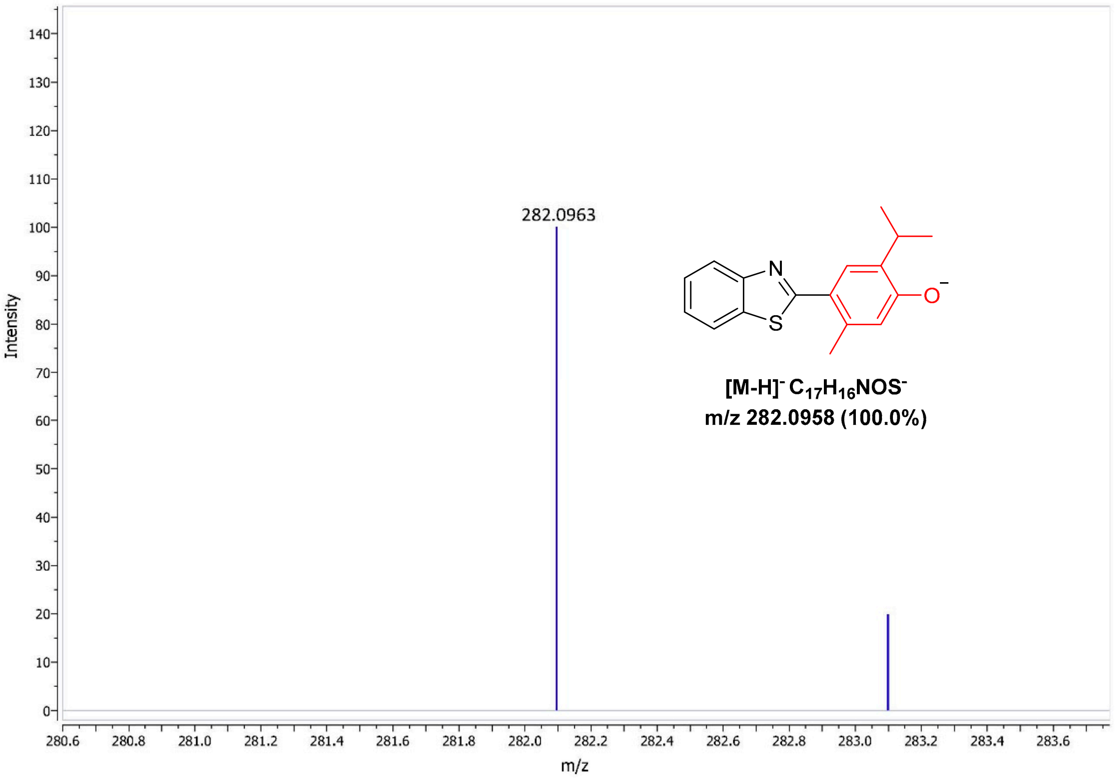

Figure S29 ¹H-NMR spectrum of **7 -** DMSO-d₆, 80 °C, 600 MHz

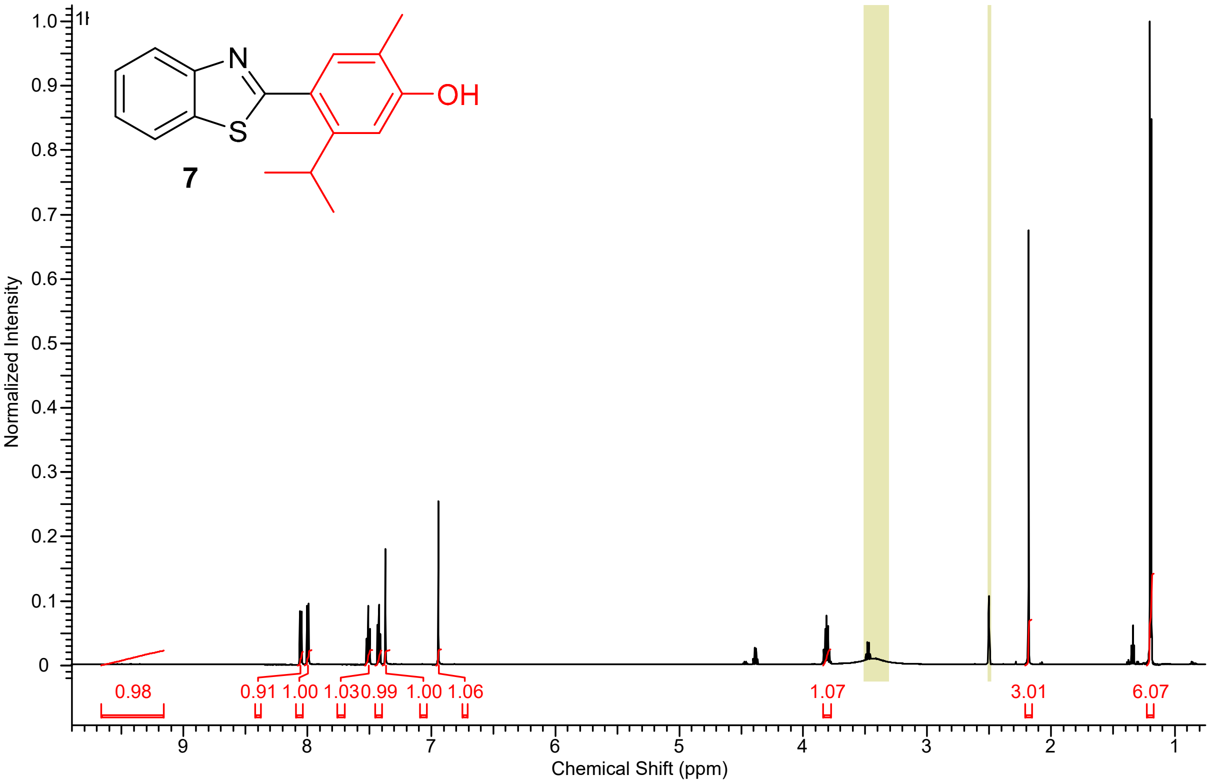

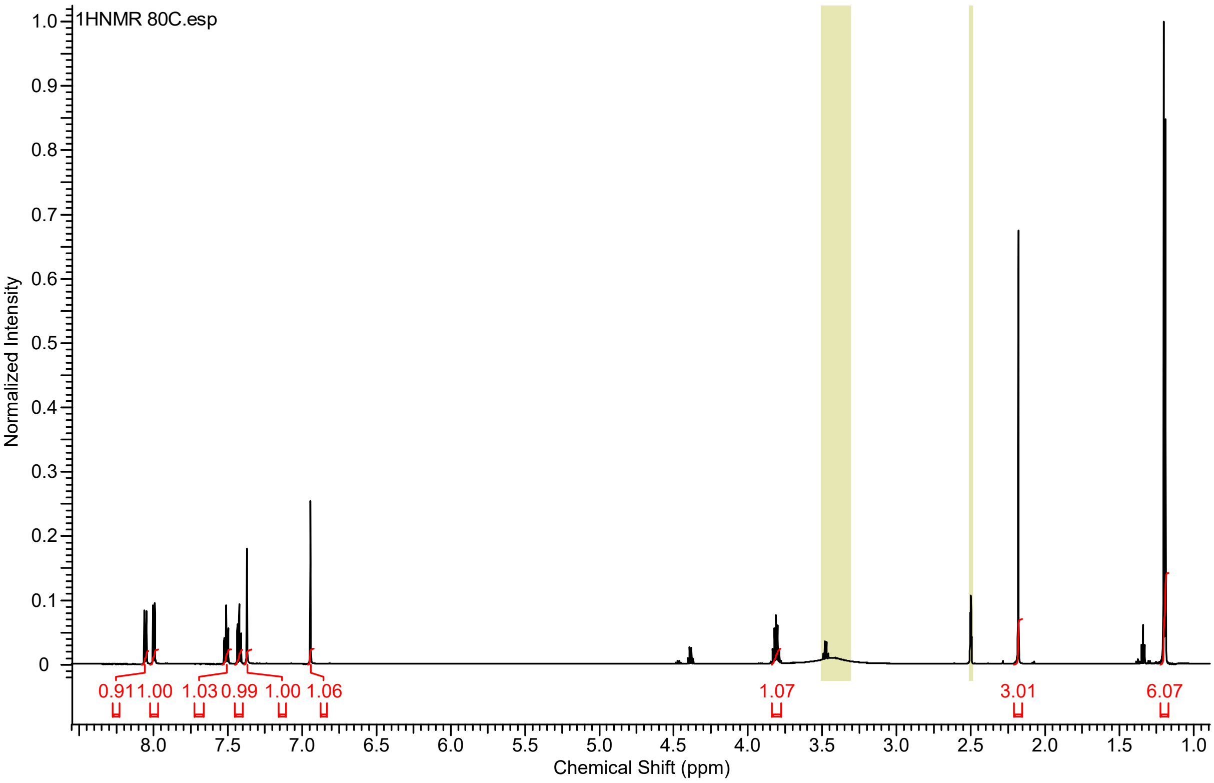

Figure S30 ^{13}C-NMR spectrum of **7 -** DMSO-d$_6$, 80 ºC, 150 MHz

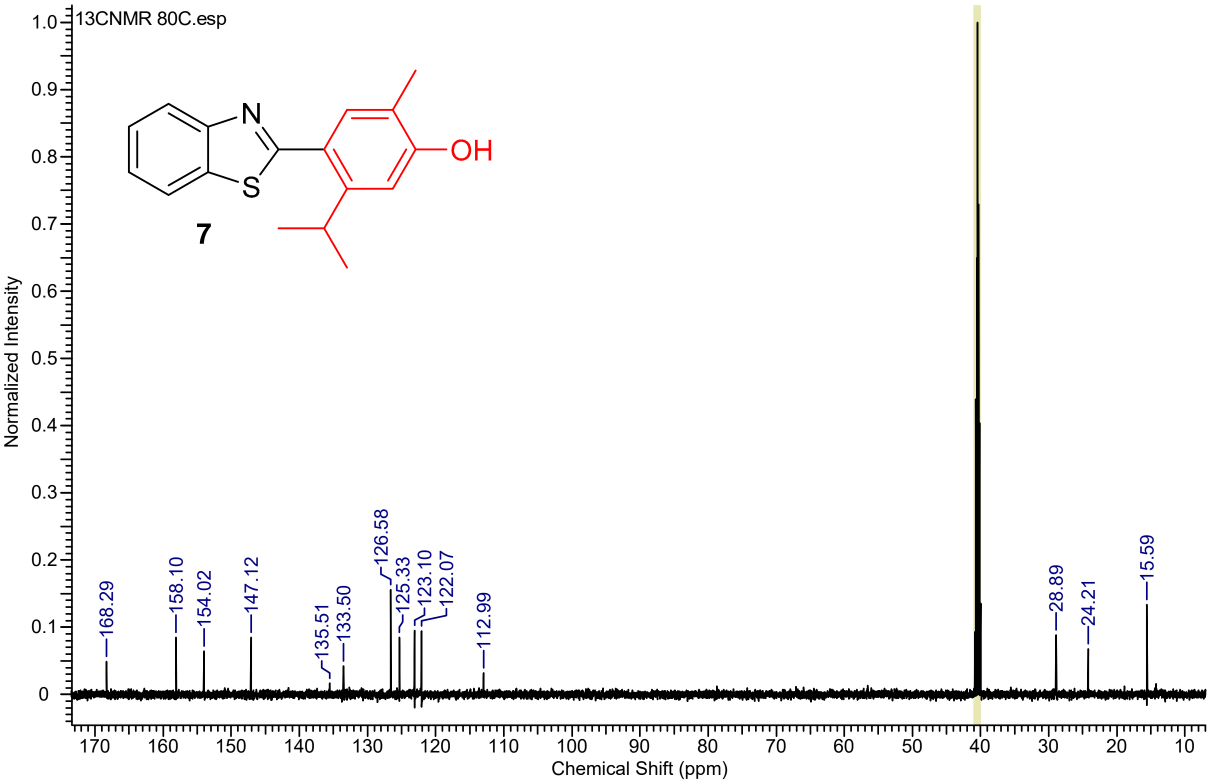

Figure S31 FT-IR spectrum of compound **7**, KBr tablet, cm^{-1}

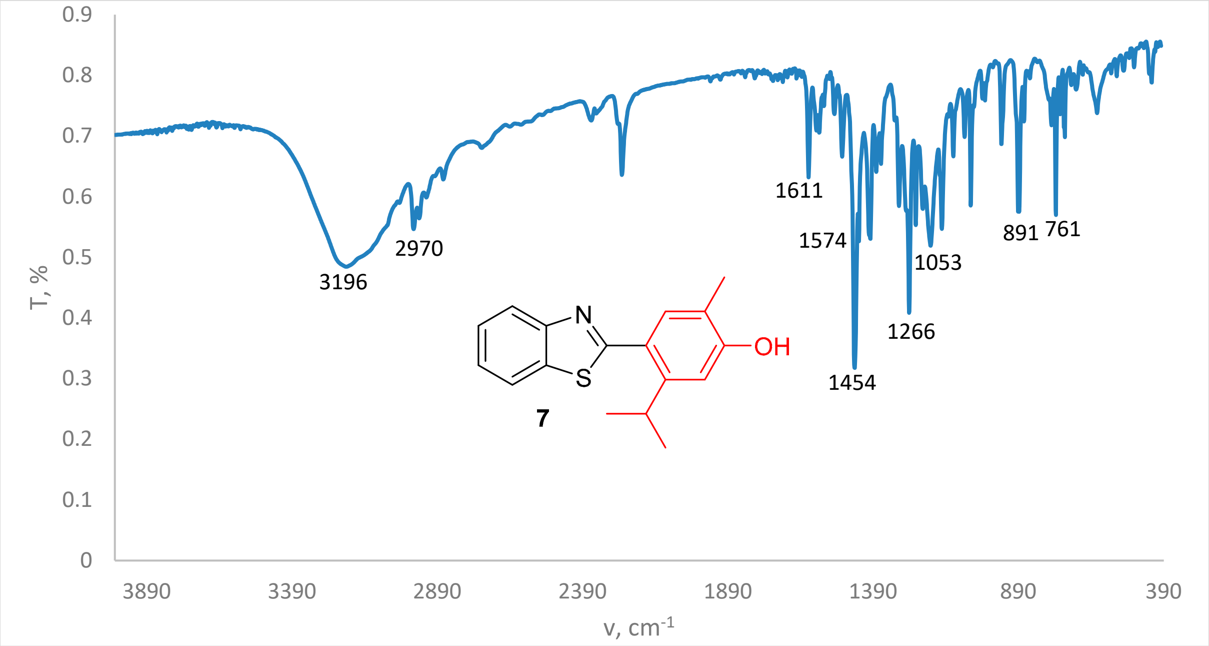

Figure S32 ESI-HRMS spectrum of compound **7**

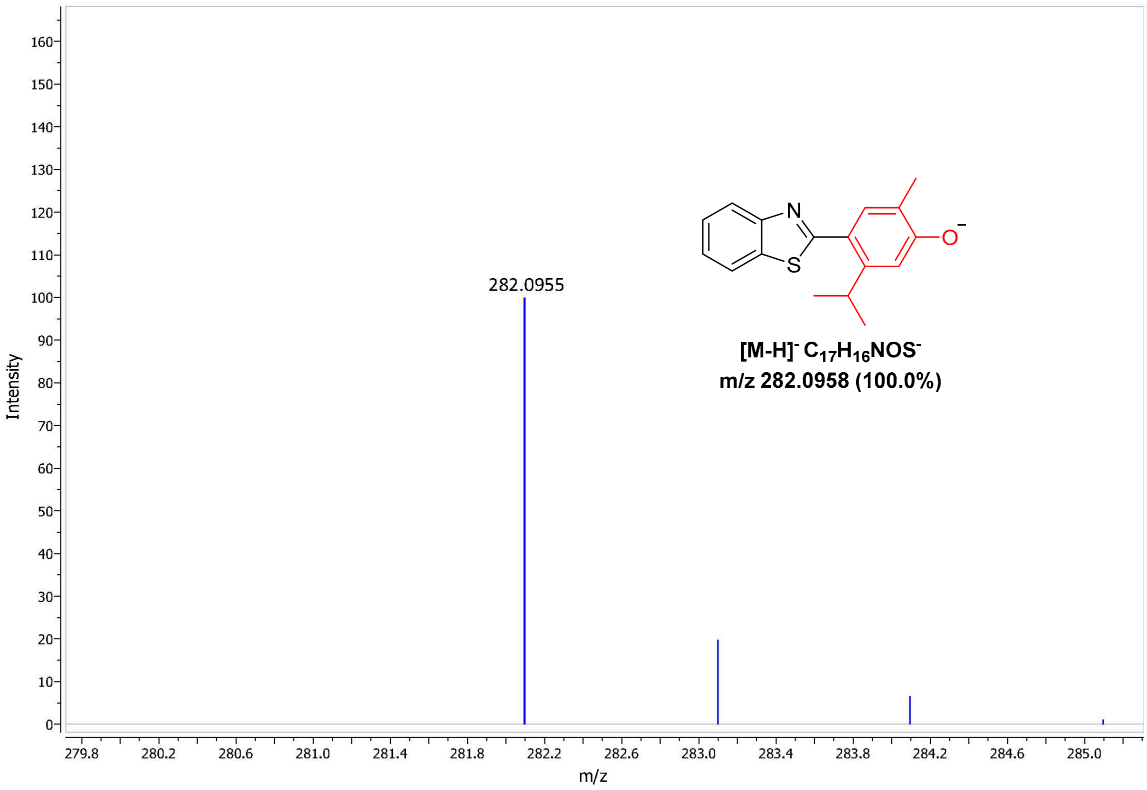

Figure S33. GC-MS/MS analysis - full scan 25-500 m/z, dwell time: 0.2 sec

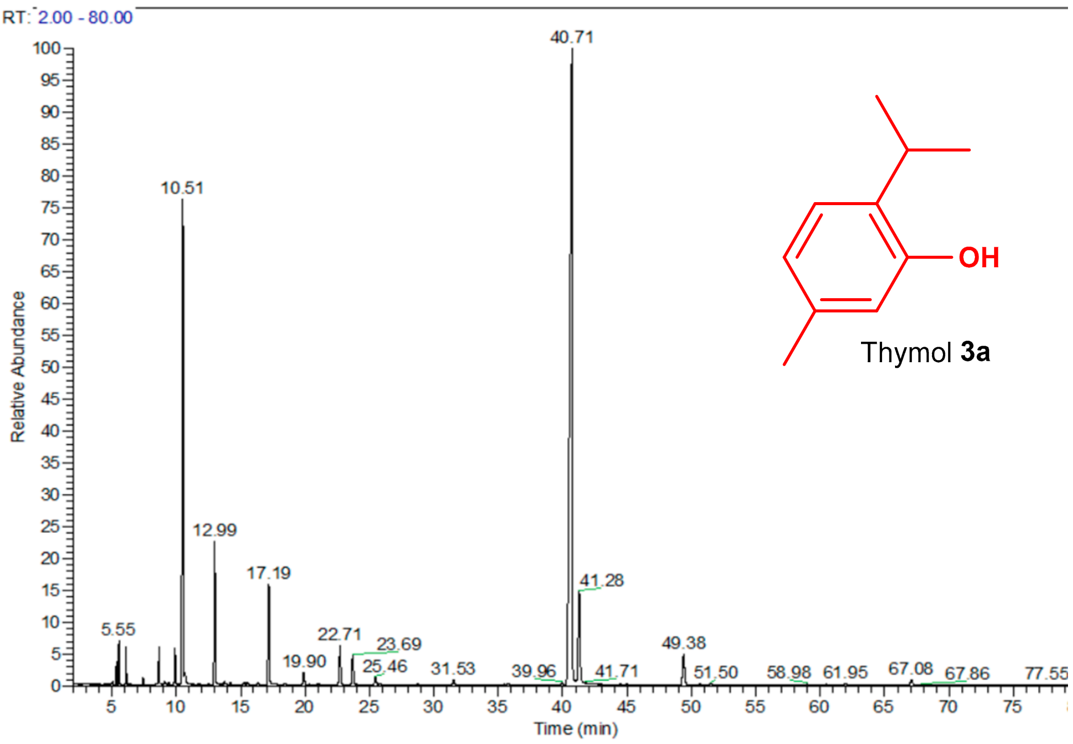

Table S1. Component composition of thyme essential oil (*Thymus vulgaris*) obtained by GC-MS/MS analysis

Apex RT	Compound	RI	Area	Area, %
**5.36**	α-Thujene	929	104434816	0.45
**5.55**	α-Pinene	929	202877822	0.87
**6.11**	Camphene	952	194880519	0.84
**7.43**	Sabinene	974	39760315	0.17
**8.65**	β-Myrcene	991	248247929	1.07
**9.10**	α-Phellandrene	1005	23801823	0.10
**9.42**	3-Carene	1011	13420274	0.06
**9.90**	α-Terpinene	1017	259788308	1.12
**10.51**	p-Cymene	1025	4084754060	17.61
**10.68**	Limonene	1030	137940130	0.59
**12.99**	γ-Terpinene	1060	1270342619	5.48
**13.74**	4-Thujanol	1075	35007920	0.15
**15.29**	Terpinolene	1088	18834109	0.08
**17.19**	Linalool	1099	1086102774	4.68
**19.90**	Camphor	1145	137948452	0.59
**22.71**	endo-Borneol	1167	475572950	2.05

**23.69**	Terpinen-4-ol	1177	351648334	1.52
**25.46**	*α*-Terpineol	1189	90410362	0.39
**31.53**	Thymol methyl ether	1235	62694657	0.27
**40.71**	**Thymol**	**1291**	**12421001540**	**53.54**
**41.28**	Carvacrol	1299	1392669846	6.00
**49.39**	Caryophyllene	1419	463930841	2.00
**67.08**	Caryophyllene oxide	1581	82353730	0.35

Figure S34. GC-MS/MS analysis - full scan 25-500 m/z, dwell time: 0.2 sec

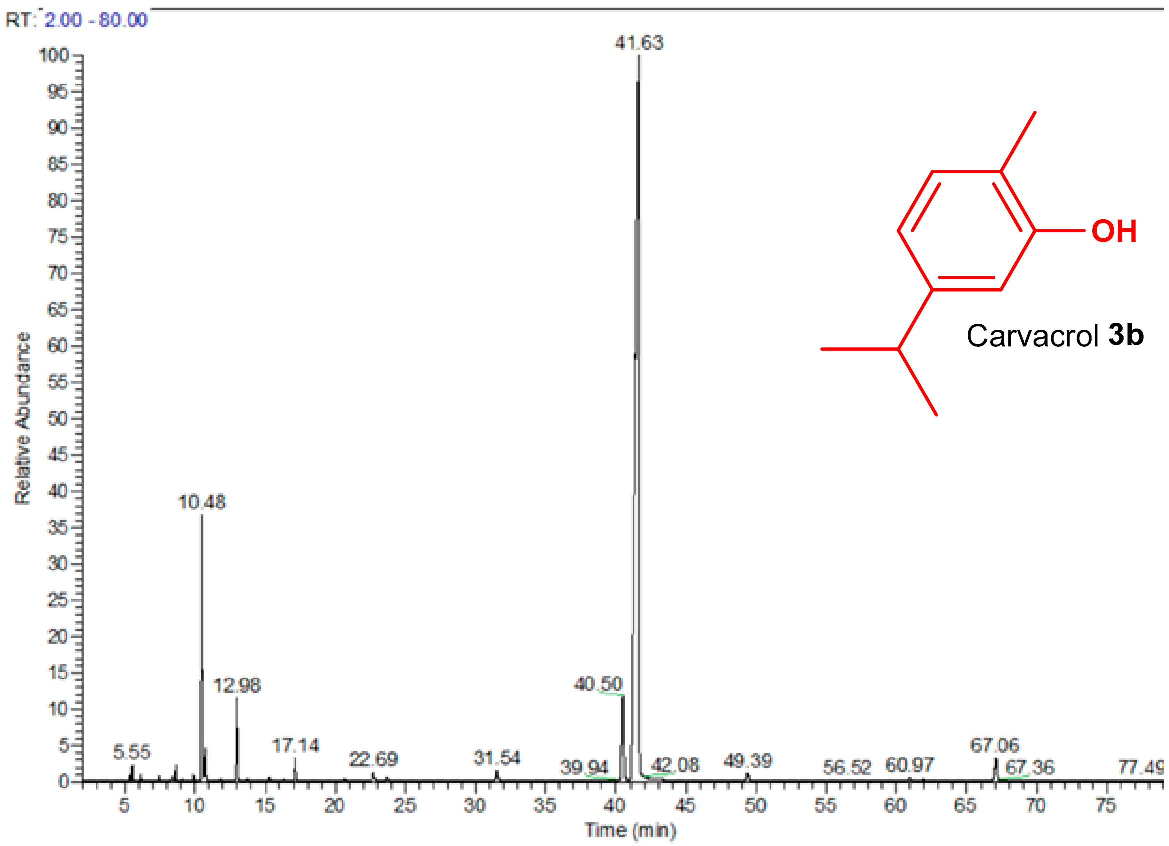

Table S2. Component composition of oregano essential oil (*Origanum vulgare*) obtained by GC-MS/MS analysis

Apex RT	Compound	RI	Area	Area, %
**5.36**	*α*-Thujene	5.44	24699853	0.10
**5.55**	*α*-Pinene	5.65	68092605	0.26
**6.11**	Camphene	6.23	30148082	0.12
**7.43**	Sabinene	7.56	30041469	0.12

**8.38**	1-Octen-3-ol	8.54	35187901	0.14
**8.65**	*β*-Myrcene	8.82	94947297	0.37
**9.07**	Pseudolimonen	9.2	7639584	0.03
**9.89**	*α*-Terpinene	10	38440427	0.15
**10.48**	*p*-Cymene	10.6	2067346229	8.01
**10.75**	Limonene	10.95	323258829	1.25
**11.83**	*β*-cis-Ocimene	11.97	10214706	0.04
**12.98**	*γ*-Terpinene	13.2	712755284	2.76
**13.74**	4-Thujanol	13.99	14044344	0.05
**15.29**	Terpinolene	15.42	25147367	0.10
**17.14**	Linalool	17.37	221496870	0.86
**22.69**	endo-Borneol	22.89	96326065	0.37
**23.68**	Terpinen-4-ol	23.89	33753936	0.13
**31.54**	Thymol methyl ether	31.77	134547185	0.52
**40.50**	Thymol	40.88	1246171282	4.83
**41.63**	**Carvacrol**	**42.32**	**20072626547**	**77.78**
**49.39**	Caryophyllene	49.63	110131374	0.43
**60.97**	*β*-Bisabolene	61.17	31943716	0.12
**61.93**	*β*-Cadinene	62.14	17936082	0.07
**67.06**	Caryophyllene oxide	67.39	359908069	1.39
